# Selection and Validation of Appropriate Reference Genes for Quantitative RT-PCR Analysis in *Rubia yunnanensis* Diels Based on Transcriptome Data

**DOI:** 10.1155/2020/5824841

**Published:** 2020-01-08

**Authors:** Shanyong Yi, Qianwen Lin, Xuejia Zhang, Jing Wang, Yuanyuan Miao, Ninghua Tan

**Affiliations:** State Key Laboratory of Natural Medicines, Department of TCMs Pharmaceuticals, School of Traditional Chinese Pharmacy, China Pharmaceutical University, Nanjing 211198, Jiangsu, China

## Abstract

Real-time quantitative polymerase chain reaction (RT-qPCR) has been widely applied in gene expression and transcription abundance analysis because of its high sensitivity, good repeatability, and strong specificity. Selection of relatively stable reference genes is a precondition in order to obtain the reliable analysis results. However, little is known about evaluation of a set of reference genes through scientific experiments in *Rubia* plants. Here, 15 candidate reference genes were selected from *R. yunnanensis* transcriptome database and analyzed under abiotic stresses, hormone treatments, and different tissues. Among these 15 candidate reference genes, *heterogeneous nuclear ribonucleoprotein* (*hnRNP*), *TATA binding protein* (*TBP*), *ribosomal protein L5* (*RPL5*), *malate dehydrogenase* (*MDH*), and *elongation factor 1-alpha* (*EF-1α*) were indicated as the five most stable reference genes by four statistical programs (geNorm, NormFinder, BestKeeper, and RefFinder). Ultimately, the validity of reference genes was confirmed by normalizing the expression of *o-succinylbenzoate-CoA ligase* (*OSBL*) and *isochorismate synthase* (*ICS*) involved in the anthraquinone biosynthesis pathway in different tissues and hormone treatments. Meanwhile, four other putative genes involved in the anthraquinone biosynthesis pathway were also normalized with the selected reference genes, which showed similar expression levels with those given by transcriptome data. This work is the first research that aims at a systematic validation on the stability of reference genes selected from *R. yunnanensis* transcriptome data and will be conducive to analyze gene expression in *R. yunnanensis* or other *Rubia* species.

## 1. Introduction

Real-time quantitative polymerase chain reaction (RT-qPCR) is a technique for precise quantification of nucleic acids by monitoring the entire PCR process using a real-time fluorescence quantifier. It also plays an extremely important role in gene expression analysis and is the most extensive method compared with the other three methods (northern blot, microarray, and high-throughput sequencing) due to its high sensitivity, good repeatability, strong specificity, high throughput, wide application, and low cost [1].

RT-qPCR is a powerful tool for understanding metabolic pathways, genetics, signaling pathways, and several complex regulatory networks in organisms. The accuracy of results using this method is unavoidably affected by sample amounts, RNA integrity, cDNA quality, efficiency of enzymatic reaction, and gDNA contamination [2, 3]. In order to obtain stable and reliable results in the RT-qPCR assay, a suitable and stable reference gene is a prerequisite to normalize gene expression and avoid errors caused by the factors mentioned above.

Reference genes are usually housekeeping genes, which are expressed in all kinds of cells in organisms, and their products are essential proteins to maintain the basic life activities of cells. There are hundreds of housekeeping genes, among which *GAPDH* (*glyceraldehyde-3-phosphate dehydrogenase*), *ACT* (*actin-related protein*), *TUB* (*beta-1 tubulin*), 18S *rRNA* (*ribosomal RNA*), and 28S *rRNA* are often used as internal reference genes for the standardization of target genes owing to their stable expression levels under different conditions and various tissues in many plant species [4, 5]. However, these reference genes have also shown some shortcomings that their expression levels vary greatly under certain factors even if they can be stably expressed in some cells or tissues under some experimental conditions in recent studies [6, 7]. Therefore, it is completely necessary to select suitable internal reference genes as the standard to study the expression levels of target genes according to the different sample types and test conditions in order to make the results more reliable. With the development in science and technology, several new statistical algorithms such as geNorm, NormFinder, BestKeeper, and RefFinder have emerged for screening stable internal reference genes in recent years, which have been developed as efficient tools to determine conveniently the most stable reference genes for RT-qPCR normalization [8–10]. Hence, many novel reference genes were validated, and their suitability was evaluated by employing these programs in different plants, such as *Euscaphis konishii* [11], *Sapium sebiferum* [12], and *Neolamarckia cadamba* [13].

*Rubia yunnanensis* Diels is a Chinese medicinal herb mainly distributed in Yunnan Province in Southwest China, and its medicinal parts are the roots and rhizomes named “Xiaohongshen,” which are used to treat many diseases in folk medicines such as cancer, vertigo, insomnia, rheumatism, tuberculosis, menstrual disorders, and contusions [14]. It can be used as a substitute in Yunnan Province for *R. cordifolia* listed in Chinese Pharmacopoeia. Three major types of important constituents, i.e., quinones, Rubiaceae-type cyclopeptides, and terpenoids, have been isolated from *R. yunnanensis* by our group [15–17]. Several of them have been reported to have important pharmacological activities, such as rubiadin [18], RA-V [19, 20], RA-XII [21, 22], and rubiarbonol G [23]. Among them, anthraquinones are the most abundant and important bioactive compounds [17], and there are still no data reported on gene expression and also about biosynthetic pathways of anthraquinones in *R. yunnanensis*. Based on previous research progress on anthraquinone biosynthesis in Rubiaceae [24, 25], we hope to increase the knowledge of anthraquinone biosynthesis in *R. yunnanensis*, particularly in the areas such as gene expression analyses and mechanisms responsible for anthraquinone biosynthesis and regulation at their enzyme and gene levels. Moreover, fewer reference genes of *Rubia* were identified through scientific and systematic experiments, and randomly selected reference genes or single gene quantification RT-qPCR assays have shown frequently variability and unreliability in gene expression under various experimental conditions [26].

In our study, 15 genes including 10 common reference genes (*GAPDH*, *eIF* (*eukaryotic translation initiation factor*), *EF-1α* (*elongation factor 1-alpha*), *UBCE* (*ubiquitin-conjugating enzyme*), *RPL5* (*ribosomal protein L5*), *TBP* (*TATA binding protein*), *TUB*, *ACT2*, *MDH* (*malate dehydrogenase*), and *PEPC* (*phosphoenolpyruvate carboxykinase*)) and 5 novel reference genes (*SAND* (*SAND family protein*), *PP2A* (*serine*/*threonine-protein phosphatase 2A*), *PTBP2* (*polypyrimidine tract-binding protein homolog 2*), *hnRNP* (*heterogeneous nuclear ribonucleoproteins*), and *F-box* (*F-box protein*)) were selected as candidate genes according to transcriptome data of the root and stem leaf (mixed stem and leaf) of *R. yunnanensis* from our laboratory (Yi et al., School of Traditional Chinese Pharmacy, China Pharmaceutical University) (unpublished data). Their expression stability in *R. yunnanensis* was evaluated by RT-qPCR across different experimental conditions including various tissues (root, stem, and leaf), abiotic stresses (osmotic stress, salt stress, oxidative stress, metal stress, and cold and heat stress), and hormone treatments referring to previous study [27]. For hormone treatments, methyl jasmonate (MeJA) and salicylic acid (SA) were used to treat plants because they could act as the important elicitors and signaling molecule and also regulate the activity of various enzymes related to secondary metabolic pathways. Optimal reference genes were confirmed for each experimental condition through four statistical algorithm programs. The results indicated that *hnRNP*, *TBP*, *RPL5*, *MDH*, and *EF-1α* were the five most stable reference genes, while *GAPDH*, *F-box*, *SAND*, *TUB,* and *PEPC* were the least stable reference genes in all sample sets. In addition, in order to confirm the suitability of selected reference genes from our results, *o-succinylbenzoate-CoA ligase* (*OSBL*) and *isochorismate synthase* (*ICS*), two critical common enzymes involving shikimate and terpenoid pathways of anthraquinone biosynthesis, were chosen for normalizing their expression levels in different tissues and different time points after MeJA treatment using the least stable genes, the two most stable genes, and their combinations as the reference genes, respectively. Finally, the relative expression levels of other four putative key genes (*isopentenyl-diphosphate delta-isomerase* (*IPPI*), *3-hydroxy-3-methylglutaryl-coenzyme A reductase* (*HMGR*), *1-deoxy-D-xylulose 5-phosphate synthase* (*DXS*), and *acetyl-CoA acetyltransferase* (*AACT*)) involved in the anthraquinone biosynthesis pathway normalized with *hnRNP* and *TBP* as reference genes were compared with those calculated by the FPKM (fragments per Kilobase Million) method [28] of transcriptome data to ensure again the reliability of selected internal reference genes.

## 2. Materials and Methods

### 2.1. Plant Material

Seeds of *R. yunnanensis* were collected legally in the wild in Kunming, Yunnan Province, China, in October 2016. After identification by Professor Heng Li from Kunming Institute of Botany, Chinese Academy of Sciences, the seeds were sown in 25 cm × 20 cm plastic pots containing a mixture of vermiculite, nutrient soil at a ratio of 2 : 1 under a set photoperiod (16 h light and 8 h dark) at 25°C, 65% relative humidity, and PAR of 30000 lux of light intensity in Kunming Institute of Botany, Chinese Academy of Sciences (25°08′N, 102°44′E). After germination, one-year-old *R. yunnanensis* seedlings were obtained and then transported to the greenhouse of China Pharmaceutical University in November 2017 (31°54′N, 118°54′E) under routine management of the greenhouse as described above. The seeds samples were deposited at Prof. Tan's lab.

The experiment was conducted in May 2018. For different tissue samples, fresh roots, stems, and leaves were harvested and frozen immediately in liquid nitrogen and stored at −80°C until RNA extraction. For drought treatment, plants were subjected to 200 mL of 25% PEG 6000 once a day for seven days. For salinity stress treatment, plants were irrigated with 200 mL of 600 mM NaCl once a day for a week. For cold and heat stress treatments, plants were placed at 4°C and 42°C for two days in the illumination incubator, respectively. For heavy metal and oxidative stress, 500 mM copper sulfate (CuSO_4_) and 50 mM hydrogen peroxide (H_2_O_2_) were applied to plant leaves once for 24 h, respectively. Exogenous H_2_O_2_ could enhance stress tolerance of many plants by activating the antioxidative system [29]. For hormone treatments, plant leaves were sprayed once with 25 mM MeJA and 5 mM SA for 6 h, respectively. Leaf samples in all of the treatments were cut with alcohol-sterilized scissors and collected in three biological replicates for further study. The plants without treatments were collected as control, and the harvested samples after treatments were washed with MILLI-Q-filtered water and frozen immediately in liquid nitrogen and stored at −80°C until RNA extraction.

### 2.2. RNA Isolation, Quality Control, and cDNA Synthesis

Total RNA was isolated from collected samples using the EASYspin Plus Complex Plant RNA kit (catalog number: RN53, Aidlab Biotechnologies Co., Ltd, Beijing, China) according to the manufacturer's instructions and treated with DNase I (catalog number: RN34, Aidlab, Beijing, China) to eliminate DNA contamination. The RNA concentration and purity were determined with a NanoDrop 2000c Spectrophotometer (Thermo Scientific, US), and RNA integrity was checked on 1% agarose gel electrophoresis. The extracted RNA had a 260/280 ratio between 1.9 and 2.1, which was considered to meet the requirements of following experiments. 0.6 *μ*g of total RNA was prepared for synthesizing first-strand of cDNA by the Goldenstar™ RT6 cDNA Synthesis kit (Tsingke, Nanjing, China) according to the manufacture's instructions.

### 2.3. Selection of Candidate Reference Genes and Primer Design

Fifteen candidate reference genes, including ten traditional reference genes (*GAPDH*, *eIF*, *EF-1α*, *UBCE*, *RPL5*, *TBP*, *TUB*, *ACT2*, *MDH,* and *PEPC*) and five novel reference genes (*SAND*, *PP2A*, *PTBP2*, *hnRNP*, and *F-box*), were selected as candidate genes to normalize RT-qPCR experiments by screening genes with relatively stable expression, which was based on their FPKM and fold change values from transcriptome sequencing data in our lab. All data on sequences and FPKM values of screened genes are shown in Additional [Supplementary-material supplementary-material-1] and Additional [Supplementary-material supplementary-material-1]. The selected candidate reference genes included *GAPDH*, *eIF*, *EF-1α*, *UBCE*, *RPL5*, *TBP*, *TUB*, *ACT2*, *MDH*, *PEPC*, *SAND*, *PP2A*, *PTBP2*, *hnRNP*, and *F-box* that have been reported to be stably expressed in many plants such as soybean [5], *Euscaphis konishii* [11], poplar [30], potato [31], flax [32], wheat [33], maize [34], foxtail millet [35], sorghum [36], pearl millet [37], and cabbage [38].

Specific primer pairs of all candidate reference genes were designed using Primer Premier 5.0 according to the following parameters: primer sequences of 18–24 nucleotides, product length of 101–259 bp, melting temperature (Tm) of 58°C–62°C, and GC content of 40%–60%. All primers were synthesized by Tsingke Company (Nanjing, China) and tested by regular PCR. The PCR products were checked by 2% agarose gel electrophoresis prior to RT-qPCR. In addition, amplification efficiency (*E*) and correlation coefficient (*R*^2^) of primer pairs were calculated by a standard curve with a 5-fold serial dilution of first-strand cDNA (0.6 *μ*g/*μ*L). The gene names, primer sequences, amplicon length, and primers Tm, *E*, and *R*^2^ of the 15 candidate reference genes and six target genes selected for validation are shown in Table 1.

RT-qPCR experiment was performed in 48-well plates on a StepOne™ Real-time PCR system (Applied Biosystems, Life Technologies, USA) using AceQ qPCR SYBR Green Master Mix (Vazyme, Nanjing, China). Total 20 *μ*L reaction volume contained 10 *μ*L of 2x AceQ SYBR Green Master Mix (high ROX premixed), 0.4 *μ*L of each primer (10 *μ*M), 2 *μ*L of cDNA template described in item 2.2, and 7.2 *μ*L of nuclease-free water. The reaction was conducted at the following conditions: predenaturation at 95°C for 5 min, followed by 40 cycles of denaturation at 95°C for 10 s, and annealing/extension at 60°C for 30 s. The melting curve was analyzed to determine primer specificity with the conditions at 95°C for 15 s, 60°C for 60 s, and 95°C for 15 s. qRT-PCR analysis was conducted with three biological and technical replicates, and all raw data are listed in Additional [Supplementary-material supplementary-material-1].

### 2.4. Data Analysis

The three most common Microsoft Excel-based statistical softwares (geNorm, NormFinder, and BestKeeper) were applied for the evaluation of the expression stability of 15 selected candidate reference genes across all the experimental sets. Expression levels of the tested genes were determined by the amplification cycle threshold (Ct) values. The raw Ct values could not be used directly by geNorm and NormFinder, which should be converted into relative quantified data “2^(−ΔCt)^.” ΔCt was calculated by the Ct value of each sample set subtracted with their minimum Ct (ΔCt = sample Ct − minimum Ct). Gene expression stability value (*M*) and pairwise variation (*V*) were calculated by geNorm according to the criterion that the smaller *M* value had the more stability, and *V* value was used to determine the optimal numbers of reference genes with a cut-off value of 0.15 [8, 39]. NormFinder was not only used to compare the expression differences of candidate reference genes based on the calculated stability value but also used to calculate the intra- and intergroup variations. Like geNorm, the smaller the *M* value, the better the stability of internal reference gene. However, the NormFinder program can only select out the most suitable internal reference gene compared to geNorm. BestKeeper was applied with the raw Ct directly to obtain standard deviations (SDs) and coefficient of variation (CV) between pairs of genes, and the criterion for gene stability was smaller CV and SD values. Ultimately, considering the differences in ranking orders of candidate reference genes given by these three different algorithms, the web-based tool RefFinder (http://leonxie.esy.es/RefFinder/) was used to rerank the candidate genes, and the results indicated that the most stably expressed gene has the lowest value [40].

### 2.5. Validation of Reference Genes and Expression Analysis of Putative Key Genes of Anthraquinone Biosynthesis Pathway

In order to evaluate the reliability of the selected reference genes, we analyzed the relative expression levels of two key enzyme-coding genes *OSBL* and *ICS* involved in the anthraquinone biosynthesis pathway in *R. yunnanensis* [24]. The expression levels of *OSBL* and *ICS* were determined and normalized using the two most and one least stable reference genes and their combinations as reference genes under various tissues (root, stem, and leaf) and MeJA treatments. For MeJA treatments, plants were sprayed once with 200 *μ*M MeJA and leaves were then taken as samples after 1 h, 6 h, 12 h, and 24 h.

To further confirm the reliability of the proposed reference genes, we selected six putative key genes all identified as differentially expressed genes (DEGs) involved in the known anthraquinone biosynthesis pathway in root and stem leaf of *R. yunnanensis*. Expression levels of these six target genes were normalized to those of *hnRNP* and *TBP*. Moreover, RT-qPCR results of these genes were compared with their relative expression levels exhibited by FPKM values of *R. yunnanensis* transcriptome data ([Supplementary-material supplementary-material-1]). The RT-qPCR experimental method was the same as described above, and the 2^−ΔΔCt^ method was used to calculate their relative expression levels. RT-qPCR data were obtained with three biological replicates and analyzed with variance (ANOVA) followed by Student's *t*-test (*P* < 0.05).

## 3. Results

### 3.1. Verification of Amplicons, Primer Specificity, and PCR Amplification Efficiency

**Figure 1:**
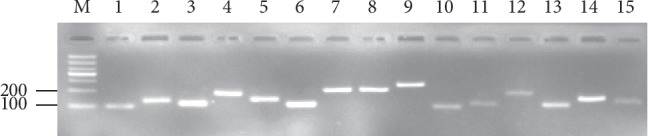
Specificity of primer pairs for RT-qPCR amplification. The 2% agarose gel electrophoresis shows the expected size of a single band for each candidate reference gene. M represents the DNA size marker. Lane 1–lane 15: *GAPDH*, *ACT2*, *EF-1α*, *eIF*, *TBP*, *TUB*, *PP2A*, *UBCE*, *SAND*, *PEPC*, *MDH*, *hnRNP*, *PTBP2*, *RPL5*, and *F-box*.

**Figure 2:**
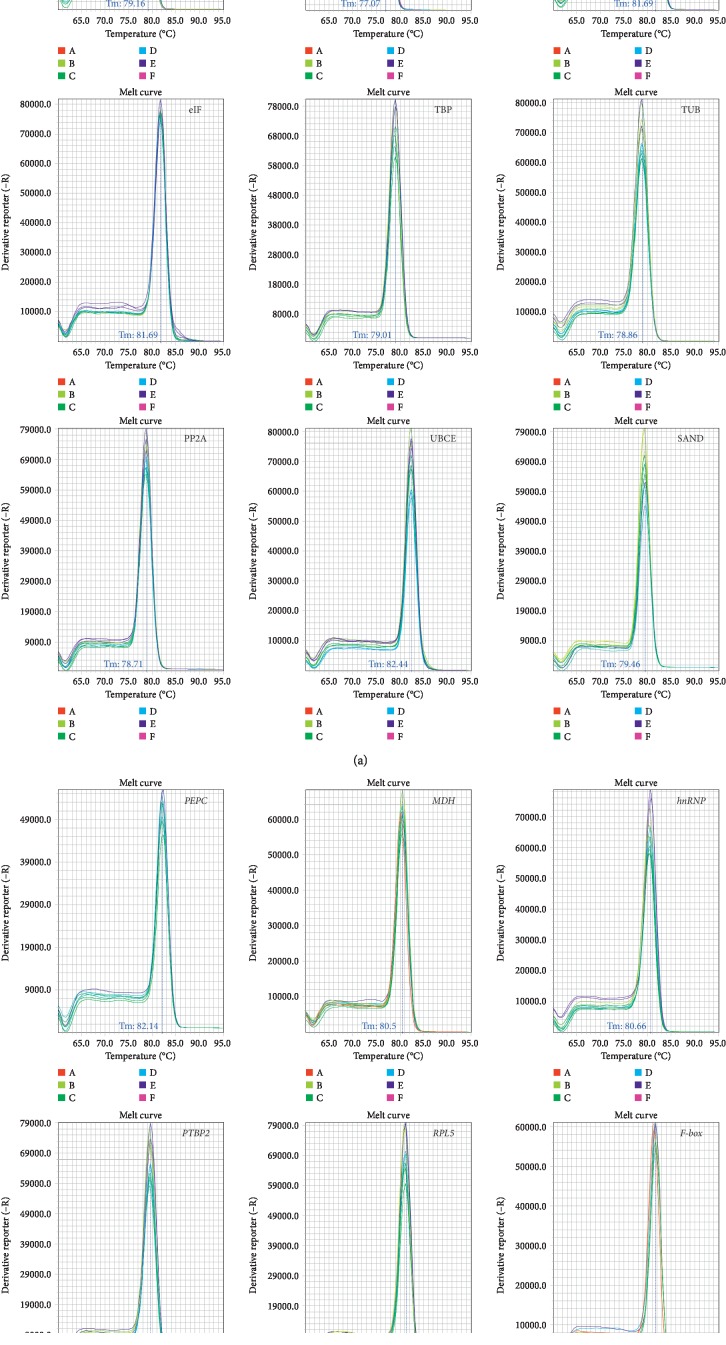
Specificity of primer pairs for RT-qPCR amplification. Melt curves with single peaks produced for all amplicons.

The specific amplification of all primer pairs of candidate reference genes was confirmed with regular PCR and RT-qPCR. A single PCR band was found with expected size by 2.0% agarose gel electrophoresis analysis (Figure 1). A single amplification peak for each candidate reference gene was observed in the melting curve (Figure 2). The PCR amplification efficiencies for these genes ranged from 93.36% for *PTBP2* to 108.08% for *UBCE*, and the correlation coefficients varied from 0.999 to 0.990 (Table 1).

### 3.2. Expression Profile of Candidate Reference Genes

**Figure 3:**
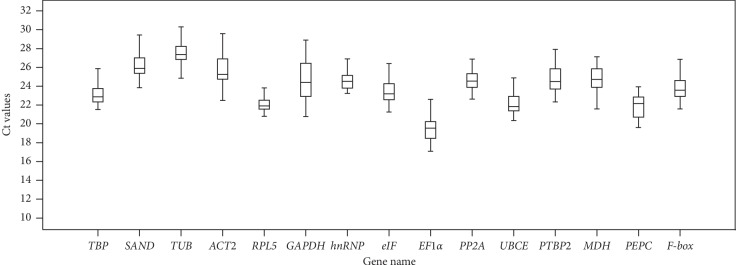
RT-qPCR raw Ct values for candidate reference genes in different samples. The line across the box depicts the median. The box indicates the 25th and 75th percentiles, and whisker caps show the maximum and minimum values. The lower the boxes and whisker, the smaller the variations.

The raw Ct values of the 15 candidate reference genes for RT-qPCR were collected and are shown in Figure 3. The Ct values varied from 17.13 (*EF-1α*) to 30.55 (*TUB*), and the average Ct values ranged from 19.40 (*EF-1α*) to 27.49 (*TUB*), which indicate that obvious differences exist in the expression profiles. Low Ct values correspond to high expression levels. Therefore, *EF-1α* exhibited the highest expression abundance and *TUB* expressed the lowest level. Moreover, Ct values also have shown the differential expression variability, and *RPL5* and *hnRNP* had a relative narrower Ct range than other genes, showing that these two genes might be expressed more stably.

### 3.3. Expression Stability of Candidate Reference Genes

In order to further confirm the stability of candidate reference genes, different treatments including osmotic stress, salt stress, heavy metal stress, oxidative stress, hormone, and temperature stress and different tissues were selected for the RT-qPCR experiments of 15 candidate reference genes. Then, 1485 Ct values (three biological and technical replicates, Additional [Supplementary-material supplementary-material-1]) were obtained for further analysis by geNorm, NormFinder, BestKeeper, and RefFinder.

#### 3.3.1. geNorm Analysis

**Figure 4:**
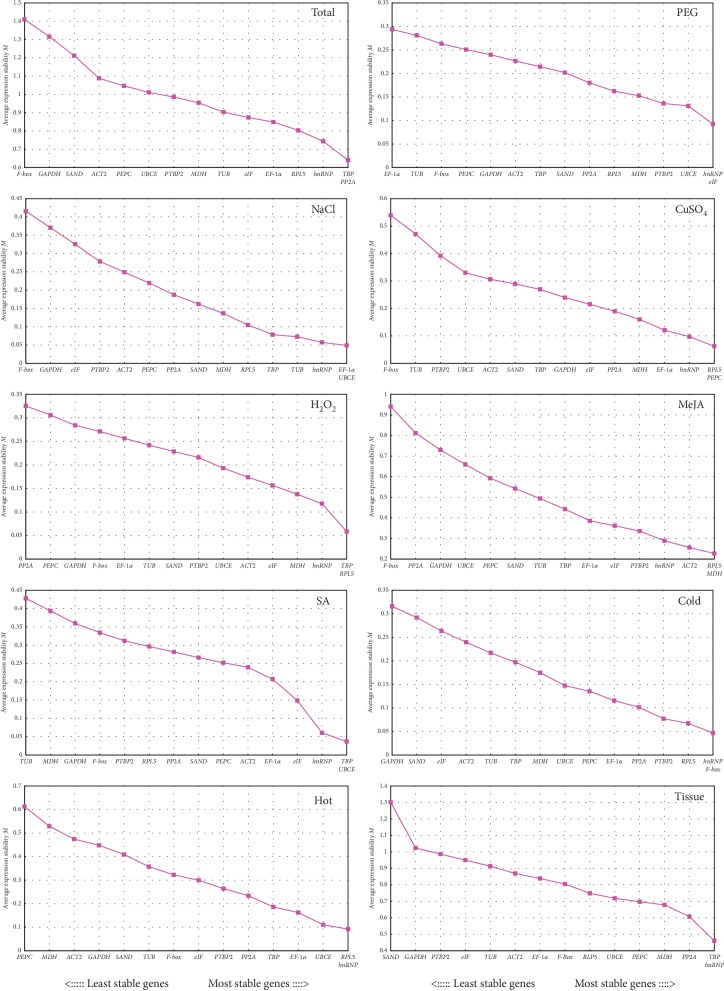
Average expression stability values *M* of 15 candidate reference genes calculated by geNorm. Total: the mixed samples of all given conditions; PEG: drought treatment; NaCl: salinity treatment; CuSO_4_: heavy metal treatment; H_2_O_2_: oxidation treatment; cold and hot: temperature treatment; MeJA and SA: hormone treatment; tissue: total tissues samples. The least stable genes are on the left and the most stable genes on the right.

In geNorm analysis, measurement (*M*) values of expression stability of the reference genes were generated as the level of pairwise variation for each reference gene with all other control genes until the most stable reference genes were identified [8]. A cut-off *M* value of 1.5 was recommended for evaluating all genes stability and a lower *M* value meant a higher stability. In our study, the *M* values of the candidate genes were all lower than 1.5 and were ranked by average expression stability values of the reference genes. As shown in Figure 4, all of the most stable reference genes in the 15 candidate genes were not identical under different treatments and tissues. The most stable genes were *hnRNP* for the groups of tissue, PEG, and cold treatments, *RPL5* for the groups of CuSO_4_, MeJA, and hot treatments, *TBP* for the groups of total (all the samples were mixed), H_2_O_2_ and SA treatments, and *EF-1α* for the NaCl group according to their lowest *M* values. Correspondingly, the least stable genes were as follows: *F-box* for the total, CuSO_4_, MeJA, and NaCl groups, *EF-1α*, *PP2A*, *PEPC*, *GAPDH*, *TUB*, and *SAND* for the PEG, H_2_O_2_, hot, cold, SA, and tissue groups, respectively.

However, an interesting finding was that the same reference genes could have different stabilities under various groups such as *EF-1α*, which was the most stable reference gene in the NaCl group but was the least stable reference gene for the PEG group. Another important information also observed was that MeJA treatment had a greater impact on plants than other treatments based on its wider *M* value (0.23 (*RPL5*) to 0.94 (*F-box*)), while PEG had the least impact with the low span of *M* value (0.09 (*hnRNP*) to 0.29 (*EF-1α*)), meaning that *EF-1α* was the least stable reference gene in the PEG group, but it could be selected as a reference gene for other experimental conditions because of its low *M* value. This phenomenon might also be used to explain why *EF-1α* could become the most stable reference gene in the NaCl group. Finally, one of the most important findings was that *hnRNP*, *RPL5*, and *TBP* seemed to be the three most stable reference genes according to their number of occurrences on the first four most stable genes in each group (*hnRNP* appeared 10 times and 6 times for *TBP* and *RPL5*, respectively).

**Figure 5:**
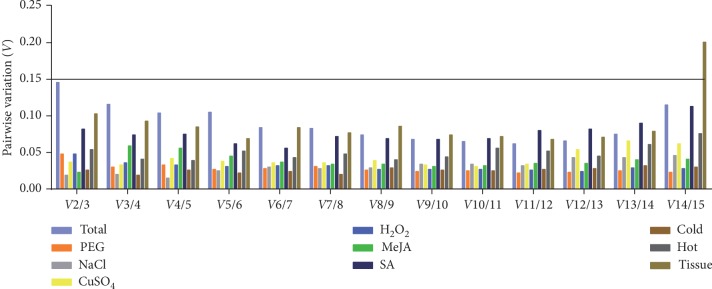
Determination of the optimal number of candidate reference genes for normalization in the *R. yunnanensis* sample sets. The pairwise variation (*Vn*/*Vn* + 1) was calculated between the normalization factors using the geNorm software program to determine the optimal number of reference genes required for RT-qPCR data normalization.

Moreover, multiple reference genes were taken into consideration for obtaining more reliable normalization results according to the calculated pairwise variation (*Vn*/*n* + 1) ratio. In order to find the appropriate number of reference genes for normalization, an ideal ratio below 0.15 was chosen for the determination on whether additional reference genes should be required [8]. According to the results of this work, two reference genes were sufficient to meet the requirements for normalization analysis of RT-qPCR due to that a pairwise variation value of *V*2/3 from each sample group was lower than 0.15 (Figure 5). The best combinations were as follows: *TBP* + *PP2A* for total samples and *TBP* + *hnRNP* for tissues, *hnRNP* + *eIF* for PEG treatment and *EF-1α* + *UBCE* for salinity stress, *RPL5* + *MDH* for MeJA treatment and *TBP* + *UBCE* for SA treatment, *TBP* + *RPL5* for oxidative stress and *RPL5* + *PEPC* for heavy metal stress, *RPL5* + *hnRNP* and *hnRNP* + *F-box* for hot and cold stress, and respectively.

#### 3.3.2. NormFinder Analysis

In order to further ensure the accuracy of experimental analysis by geNorm, NormFinder was used for analyzing expression stability values of the reference genes. Similar to geNorm, the raw data from RT-qPCR could not be used directly and should be transformed [9]. However, the difference between NormFinder and geNorm was that the former consider intra- and intergroup variations for calculating normalization factors using ANOVA to evaluate expression stability. A variation value of each reference gene was given by NormFinder, and lower values also indicate better stability (Table 2). The most stable genes could be found directly under different experimental conditions. *hnRNP* was the most stable gene with the minimum variation in PEG, CuSO_4_, H_2_O_2_, MeJA, cold treatment groups, and tissue samples, while *RPL5*, *EF-1α*, *eIF*, and *TBP* were the most stable genes for hot, NaCl, SA stresses, and total samples, respectively.

#### 3.3.3. BestKeeper Analysis

Unlike geNorm and NormFinder, the raw Ct data were used directly without conversion by BestKeeper, which compared the expression differences in samples, rather than pairwise comparisons for analyzing the expression stability of the candidate reference genes. Standard deviations (SDs) and coefficient of variation (CV) between pairs of genes were obtained to evaluate the stability of reference genes in each experimental group by BestKeeper. The most stable reference gene was considered with the lowest CV and SD, and these values should be invalid and abandoned if the value of SD was found to be greater than 1. As shown in Table 3, CV ± SD values were calculated and ranked by BestKeeper and the stability of the reference genes was gradually decreasing from the top to the end in the table similar to NormFinder. Besides, ten reference genes in group “Total” and eight genes in group “Tissue” were above the cut-off value of 1.0 and could not be applied, while all SD values of other eight groups were under 1.0, except one gene in the PEG group (*F-box*), two in the CuSO_4_ group (*TUB* and *PTBP2*), four in the MeJA group (*TUB*, *F*-*box*, *PEPC*, and *PP2A*), and four in the hot group (*SAND*, *ACT2*, *GAPDH*, and *MDH*). Because of the different analytical principles, the most stable reference gene evaluated by BestKeeper differed somewhat from the results of geNorm and NormFinder. In addition, the top five most stable reference genes in each group from BestKeeper also had some relatively consistent rankings with geNorm and Normfinder, especially *hnRNP*, *RPL5*, and *TBP* appeared with the most frequency. Unexpectedly, the top two most unstable genes analyzed by the three softwares might be unified and confirmed in different sample sets such as *F-box* and *GAPDH* for the total group, *PEPC* and *GAPDH* for the PEG group, *GAPDH* and *eIF* for the NaCl group, *TUB* and *PTBP2* for the CuSO_4_ group, *GAPDH* and *EF-1α* for the H_2_O_2_ group, *F-box* and *PP2A* for the MeJA group, *MDH* and *GAPDH* for the SA group, *GAPDH* and *SAND* for the cold group, *MDH* and *ACT2* for the hot group, and *SAND* and *eIF* for the tissue group.

#### 3.3.4. RefFinder Analysis

Taking into account the discrepancy on stability analysis among three different analytical programs, RefFinder program was applied for reanalyzing the 15 reference genes based on the geometric mean of their attributed weight, which used geNorm, Normfinder, BestKeeper, and the comparative ΔCt method to rank and compare candidate reference genes [41] (Table 4). The ranking order of the top five most stable and unstable reference genes obtained by RefFinder was broadly in consistent with the results provided by geNorm and NormFinder and had slight differences with the results from BestKeeper. For instance, under all given experimental conditions, the top two most unstable genes generated by RefFinder appeared in the top five least stable genes yielded by geNorm, Normfinder, and BestKeeper. Ranking orders of the stability of 15 reference genes were summarized in order to better observe the rankings of the analysis results from the four softwares (Additional [Supplementary-material supplementary-material-1]).

### 3.4. Validation of Reference Genes and Expression Analysis of Putative Key Genes of Anthraquinone Biosynthesis Pathway

**Figure 6:**
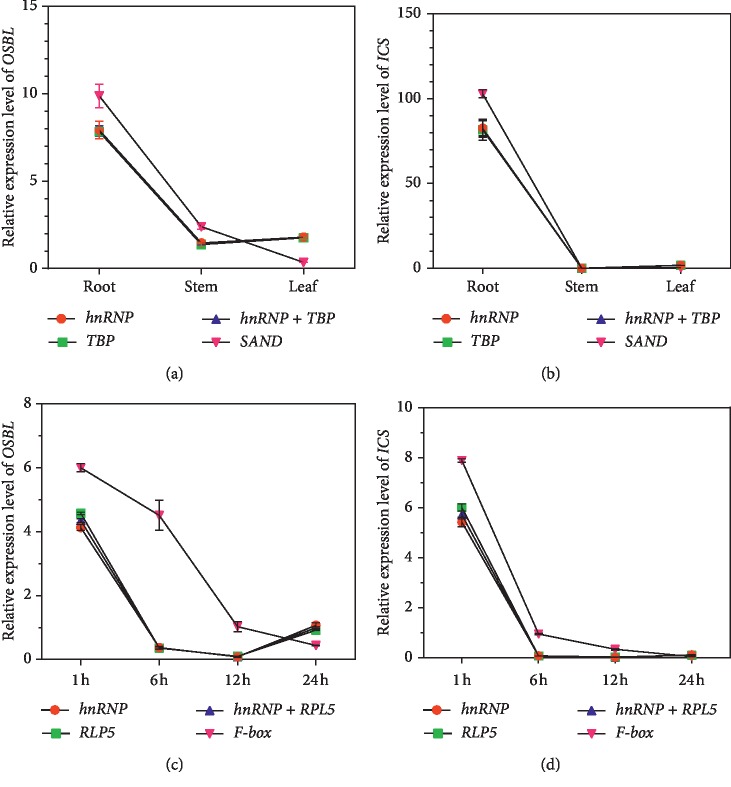
Relative expression levels of *OSBL* and *ICS* using selected reference genes (the most or the least stable reference genes) for normalization under different tissues (root, stem, and leaf) and MeJA stress treatment sets: (a) *OSBL* expression levels on different tissues; (b) *ICS* expression levels on different tissues; (c) *OSBL* expression levels on leaves under MeJA treatment after 1 h, 6 h, 12 h, and 24 h; (d) *ICS* expression levels on leaves under MeJA treatment after 1 h, 6 h, 12 h, and 24 h. The error bars represent the mean of three biological replicates ± SD.

To confirm the stability of reference genes in this study, two key enzyme-coding genes *OSBL* and *ICS* involved in the anthraquinone biosynthesis pathway in Rubiaceae [24] were selected for analyzing the relative expression levels under different tissues and MeJA treatments with two unstable reference genes (*SAND* and *F-box*), three stable reference genes (*hnRNP*, *TBP*, and *RPL5*), and their combinations as reference genes according to the comprehensive rankings of reference genes by four software programs mentioned above. As depicted in Figures 6(a) and 6(b), slight differences were produced in the relative expression levels of both *OSBL* and *ICS* when *hnRNP*, *TBP,* and *hnRNP* + *TBP* were used for the normalization in the group of different tissues. However, when using *SAND* as the normalization factor, *OSBL* and *ICS* exhibited significant differences in their relative expression levels of root and leaf samples compared with those normalized by the other three genes. The expression levels of *OSBL* and *ICS* were the highest in root, followed by leaf, and lowest in stem. For MeJA treatments (1 h, 6 h, 12 h, and 24 h), differences also emerged in the relative expression levels of both *OSBL* and *ICS* at each corresponding time point when normalized with *hnRNP*, *RPL5*, and *hnRNP* + *RPL5*. Nevertheless, extremely significant differences were observed when using the most unstable gene *F-box* as a reference gene for normalizing the relative expression levels of *OSBL* and *ICS* in 1 h, 6 h, 12 h, and 24 h samples, respectively (Figures 6(c)–6(d)). Therefore, incorrect results would be obtained when using the improper reference gene for the normalization of target genes.

**Figure 7:**
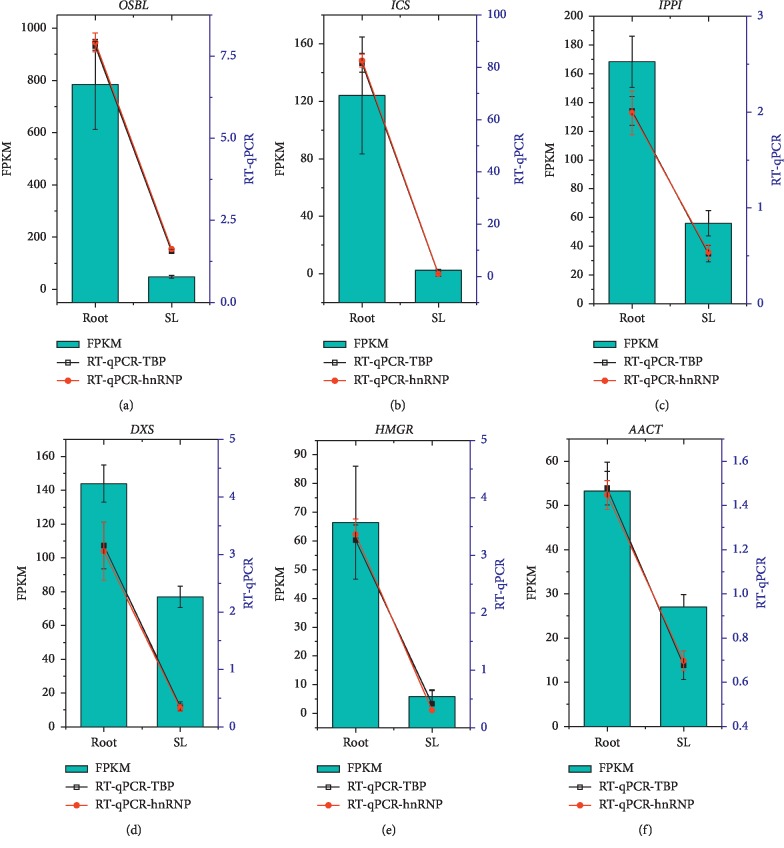
RT-qPCR validation of relative expression levels of six putative key genes involved in anthraquinone biosynthesis pathways using *hnRNP* and *TBP* as reference genes in the root and stem leaf (SL) of *R. yunnanensis*. Columns indicate relative expression levels of six genes calculated by the FPKM method (left *y*-axis) (Additional [Supplementary-material supplementary-material-1]); lines indicating the relative expression levels were obtained by RT-qPCR (right *y*-axis) (2^−ΔΔCt^ method). Error bars indicate the standard deviation of mean values of three repeats.

In our study, six putative key genes involved in the anthraquinone biosynthesis pathway were analyzed. The KEGG enrichment analysis (ko00130) showed that anthraquinones could be biosynthesized by two pathways consistent with the previous reports [24]. To better determine the accuracy and applicability of the selected reference genes and understand the expression patterns of key genes in the anthraquinone biosynthesis pathway, *hnRNP* and *TBP* were selected as reference genes for RT-qPCR data normalization of relative expression levels of six putative key genes involved in the anthraquinone biosynthesis pathway in the root and stem leaf of *R. yunnanensis* (Table 5; Additional [Supplementary-material supplementary-material-1] and [Supplementary-material supplementary-material-1]). The RT-qPCR and FPKM results were compared and presented in Figure 7, revealing that the relative expression levels of six target genes were consistent with differential expression patters in the root and stem leaf of *R. yunnanensis* and their expression levels in root were higher than those in stem leaf.

## 4. Discussion

Our current research on *R. yunnanensis* has been focused in the fields of the extraction and separation of chemical compositions and pharmacological activities [15–17, 19–23] but still not focused on its molecular function and gene expression. Therefore, *de novo* transcriptome sequencing of *R. yunnanensis* had been completed in our group for further studies concerning about the biosynthesis of secondary metabolites and related gene function mining (unpublished). To obtain accurate and reliable results for the realization of these goals, a stable and suitable reference gene would be selected and evaluated for the normalization of gene expression analysis by RT-qPCR in our research.

An ideal reference gene should be stably expressed in all samples under a range of given experimental conditions to ensure the accuracy and reproducibility of measurement of target genes expression abundance. However, no universally applicable reference genes with an invariant expression are available, which made it particularly important to screen stable reference genes. Indeed, the 15 candidate reference genes showed a relatively wide range of expression profiles in our study, confirming again that no single gene could be used for normalization in all the samples of different tissues and various experimental conditions, similar to the results in *Euscaphis konishii* [11], *Pennisetum glaucum* [37], *Bixa orellana* [42], and *Klebsiella pneumoniae* [43]. Owning to no reports on reference genes of *R. yunnanensis*, 15 candidate reference genes including 10 general housekeeping genes (*GAPDH*, *eIF*, *EF-1α*, *UBCE*, *RPL5*, *TBP*, *TUB*, *ACT2*, *MDH*, and *PEPC*) and 5 novel genes (*SAND*, *PP2A*, *PTBP2*, *hnRNP*, and *F-box*) were selected from the *R. yunnanensis* transcriptome database for evaluation according to some literatures [44–46]. RT-qPCR is considered the most common method for gene expression analyses based on its high sensitivity and specificity. The primer pairs of 15 candidate reference genes were validated and presented high specificity by regular PCR gel electrophoresis and RT-qPCR melting curve. The correlation coefficient (*R*^2^ > 0.99) (Additional [Supplementary-material supplementary-material-1]) and PCR efficiency (93.36% to 108.08%) (Table 1) indicated that the curves had a good linear relationship and the amplification conditions were acceptable. Therefore, the standard curve and amplification efficiency of each prime pair meet the requirements of the following experiments, which proved that the designed primers for 15 candidate reference genes were suitable for screening and evaluating the reference genes in *R. yunnanensis*.

The raw Ct data were obtained by RT-qPCR under the different treatments and tissues and analyzed using four most popular computational programs of gene expression analysis (geNorm, NormFinder, BestKeeper, and RefFinder) for ranking the reference genes. Different rankings of the 15 candidate reference genes were shown according to the four algorithms, indicating the necessity of utilizing more than one program to obtain the best results. Firstly, the expression levels of the candidate genes could be observed directly by the mean Ct values (Figure 3), and the lower values indicated higher expression levels. A prerequisite for accurate normalization of genes was the expression level with a suitable range of Ct values [13], and Ct values ranged from 17.13 (*EF-1α*) to 30.55 (*TUB*) in this study were sufficient for experimental needs (Additional [Supplementary-material supplementary-material-1]). The stability of genes could be reflected in the ranges of different transcriptional abundances from candidate reference genes. For instance, the expression of *RPL5* and *hnRNP* might be more stable than others according to the relative narrower Ct range. Importantly, these results were somehow consistent with the outcomes calculated by geNorm, NormFinder, BestKeeper, and RefFinder (Figure 4 and Tables 2–4), indicating that the expression level and the stability analysis of reference genes need to be combined with each other. Moreover, these results also revealed that none of 15 reference genes could be expressed constantly in all different conditions and tissues from *R. yunnanensis.*

For different analysis softwares, geNorm evaluated the stability of reference genes with the average pairwise variation [8], and NormFinder exhibited the expression stability of reference genes by analyzing their intra- and intergroup variation. However, the CV and SD values were the key factors in determining the stability rankings of reference genes obtained by BestKeeper [10]. Thus, the results were also reasonable for some differences in the ranking order shown by the three programs in our research. In geNorm analysis, *hnRNP*, *RPL5*, *TBP*, and *EF-1α* were the most stable genes and *F-box*, *EF-1α*, *PP2A*, *PEPC*, *GAPDH*, *TUB*, and *SAND* were the least stable genes under different conditions, which was relatively consistent with the results given by NormFinder, and thus further ensured the accuracy and reliability of the analysis results.

However, *F-box*, the most unstable reference gene in NaCl and CuSO_4_ groups from geNorm and NormFinder analyses, was ranked at a top position in BestKeeper analysis. Similar research findings were reported in some literatures on the selection and validation of reference genes in other plant species such as sorghum [36] and pearl millet [37]. Fortunately, BestKeeper still showed some conformance in the top five most stable reference genes with geNorm and Normfinder (Figure 4; Tables 2 and 3). Finally, a widely used web-based tool RefFinder was used to obtain a consensus stability ranking of each candidate gene under the given conditions according to the geometric mean of the attributed weights of each gene [45, 47]. For RefFinder analysis, *hnRNP*, *RPL5*, *TBP*, and *EF-1α* were ranked as the top four most stable reference genes similar with the results from geNorm, Normfinder, and BestKeeper. Synthetically, *hnRNP*, *TBP*, *RPL5*, *MDH*, and *EF-1α* were the five most frequent and stable genes ranked by the four programs with *hnRNP* occurring for 37 times, *TBP* for 26 times, *RPL5* for 25 times, *MDH* for 18 times, and *EF-1α* for 17 times. Among them, the top three stable genes given by BestKeeper were *hnRNP* for 8 times, *TBP* for 5 times, and *RPL5* for 4 times. Correspondingly, the top five least stable genes in all groups were also obtained for *GAPDH* with 36 times, *F-box* with 20 times, *SAND* with 19 times, *TUB* with 17 times, and *PEPC* with 16 times. Noticeably, the most unstable gene seemed to be determined from the top five least stable genes in each group (Additional [Supplementary-material supplementary-material-1]).

Based on the stability value sequence, *hnRNP*, *RPL5*, and *TBP* ranked firstly and were considered the most stable reference genes for analyzing all sample sets. Traditional housekeeping genes have been used as internal reference genes for normalization for a long time because proteins encoded by housekeeping genes were either used to maintain the cell structure or to participate in basic cellular metabolic processes. Theoretically, they could be stably expressed in different types of cells or in different physiological states. However, previous reports have demonstrated that some housekeeping genes have shown poor expression stability in some experimental conditions, which could not be used as internal reference genes for RT-qPCR analysis [48, 49]. In this study, the traditional housekeeping genes *RPL5*, *TBP*, *MDH*, and *EF-1α* exhibited quite good stability under the given conditions. Unexpectedly, a novel reference gene, *hnRNP*, vital for the translation of mRNA in the cytoplasm [50] also showed particularly excellent stability in all sample sets. Furthermore, increasing studies in various species [51–53] tended to apply multiple references rather than a single one considering the reliability and accuracy for the normalization of RT-qPCR data. In our study, the combination of two reference candidate genes has already satisfied the normalization under all the experimental conditions and various tissues (Figure 5).

To confirm the suitability of the reference genes selected from our study, a validation experiment is prerequisite for the evaluation of the reference genes. Three stable reference genes (*hnRNP*, *TBP*, and *RPL5*) and their combinations and two of least stable genes (*SAND* and *F-box*) were used for the normalization of *OSBL* and *ICS*. The relative expression levels of *OSBL* and *ICS* involved in anthraquinone biosynthesis were assessed under different tissues and MeJA treatments. Apparently, only the slight differences were noticed in the relative expression levels of *OSBL* and *ICS* between both the groups of different tissues and MeJA treatments when using the pairs of selected stable reference genes alone or their combinations for normalization. However, the opposite results were exhibited when the least stable reference genes were used for the normalization of relative expression levels of *OSBL* and *ICS* between different tissues and MeJA treatments, suggesting that the selection and confirmation of suitable and stable reference genes were particularly critical for the proper normalization for the RT-qPCR data in *R. yunnanensis.* Besides, significant differences were found in which the relative expression levels of *OSBL* and *ICS* in root were much higher than those on stem and leaf (Figures 6(a) and 6(b)).

These results might be regarded as a reference to reflect the contents of anthraquinones in *R. yunnanensis* and used to explain why the root of *R. yunnanensis* is used as the major medicinal parts. For the MeJA stress, the relative expression levels of *OSBL* and *ICS* were markedly upregulated after *R. yunnanensis* was treated for 1 h and then significantly downregulated in 6 h and 12 h until they tend to rise slowly again between 12 and 24 hours, indicating that some strong responses were produced and enhanced in the relative expression levels of *OSBL* and *ICS* of *R. yunnanensis* at the beginning of the MeJA stimulation for 1 h (Figures 6(c) and 6(d)). This phenomenon might be elucidated by previous literature reports indicating that MeJA plays critical roles in the increase of anthraquinone contents in Rubiaceae plants by upregulating the expression levels of genes related to anthraquinone biosynthetic pathways [24, 54]. It is also known that MeJA could upregulate the expression of defense-related genes [55], so the upregulation of *OSBL* and *ICS* in response to MeJA treatment on *R. yunnanensis* might indicate that anthraquinone biosynthesis is possibly related to its defense response.

Anthraquinones are the most abundant and important bioactive compounds, and their biosynthesis pathway is still unclear in *R. yunnanensis*. However, major routes of biosynthesis of anthraquinones in Rubiaceae have been reported that its rings A and B are formed via the shikimate pathway and ring C for the terpenoid pathway [24]. In anthraquinone biosynthesis, we selected the six putative key DEGs encoding anthraquinone biosynthetic enzymes based on KEGG enrichment pathway analysis from the transcriptome database of *R. yunnanensis* and previous literature report [24]. A further comparison was conducted with the RT-qPCR and FPKM results on relative expression levels of six target genes in the root and stem leaf of *R. yunnanensis*. These two-part results both showed the similar differential expression patterns of six target genes in the root and stem leaf of *R. yunnanensis*, indicating that *hnRNP* and *TBP* could be indeed stable reference genes in the tissue group (Figure 7). The higher expression levels of these target genes in the root than stem leaf might indicate that root was the main organ for anthraquinone synthesis. Consequently, the results of our analysis not only highlighted the importance and necessity of selecting suitable and stable reference genes for the quantitative experiments by RT-qPCR but also established a foundation to explore the biosynthetic pathway of anthraquinones.

## 5. Conclusions

Our study presented the evaluation of the expression stability of 15 candidate reference genes, including 10 common housekeeping genes and 5 novel genes obtained from the transcriptome data in *R. yunnanensis* under a wide range of experimental conditions. According to our results, comprehensive rankings order was showed by four widely used programs (geNorm, NormFinder, BestKeeper, and RefFinder), indicating that *hnRNP*, *RPL5,* and *TBP* displayed better stability in all groups. Meanwhile, unstable genes were relatively consistent combining the two most unstable genes by RefFinder with the top four least stable genes by three other softwares under the corresponding conditions. In addition, the expression analysis of *OSBL* and *ICS* emphasized the importance of selecting suitable and stable genes for the normalization of gene expression analysis by RT-qPCR. Meanwhile, the expression patterns of putative key genes in anthraquinone biosynthesis pathway of *R. yunnanensis* were investigated by RT-qPCR for the first time. This study is the first report on the selection and validation of reference genes and will supply an important foundation for the future research on gene expression analyses by RT-qPCR in *R. yunnanensis* and related species of *Rubia.*

## Figures and Tables

**Table 1:** Primers used for RT-qPCR normalization.

Gene abbreviation	Gene name	Primer sequence (5′–3′)	Amplicon length (bp^*∗*^)	Primers Tm^*∗*^ (°C)	*E* ^*∗*^ (%)	*R* ^2^^*∗*^
*GAPDH*	Glyceraldehyde-3-phosphate dehydrogenase	For: GCTGAGATTCTTGATGGGGAG	101	58.7/58.5	97.90	0.998
		Rev: CTTGTGGTAGGATTTACTATGGCA				
*eIF*	Eukaryotic translation initiation factor	For: TCAGTTTACTCGTGGGACATCG Rev: GGTTCACATACGCAGCCTCG	181	60.1/61.3	93.58	0.992
*EF-1α*	Elongation factor 1-alpha	For: GAGGTTTTGAGGCTGGCATT	117	59.2/59.9	102.78	0.995
		Rev: GGGTGGTGGCATCCATCTT				
*SAND*	SAND family protein	For: GCAACTGAATCCACAGAGCG	259	58.9/59.4	94.18	0.998
		Rev: CCCAGCAAAGCAGAACATAATC				
*PP2A*	Serine/threonine-protein phosphatase 2A	For: TTGGTTCTTCGCAGTTGATTG	208	58.8/59.0	103.91	0.995
		Rev: AGCCCGCTATTGACCTTGTT				
*hnRNP*	Heterogeneous nuclear ribonucleoprotein	For: GCAAAGGCAGCACTGTCAAG	187	59.2/61.1	99.36	0.996
		Rev: CGGTAATGTTCCCGTATTTCTCA				
*UBCE*	Ubiquitin-conjugating enzyme	For: CCCAATCCGTCTGACCCTT	203	59.3/61.7	108.08	0.99
		Rev: GCTTTTCCAGCAACTTCCTCG				
*RPL5*	60S ribosomal protein L5	For: TGGTGGGCTTGACATTCCT	150	58.2/61.5	93.62	0.994
		Rev: CGCTCTGGCTCATCTTCTTCC				
*PTBP2*	Polypyrimidine tract-binding protein homolog 2	For: GGAGCAAATCGTAACCAAGCA	115	60.4/59.0	93.36	0.999
		Rev: AGACAGTTTTCCCTCGCACC				
*TBP*	TATA binding protein	For: GGGCTTGCTTACTCTCACGG	146	59.9/58.4	101.57	0.996
		Rev: TCCTCCCTAACCTTTGCTCC				
*TUB*	Beta-1 tubulin	For: CTCTTCAAGTAGGTCAATGTGGG	116	58.3/58.4	98.83	0.995
		Rev: CCCTCCCTGAGTAGCGAAGT				
*ACT2*	Actin-related protein 2	For: ATTGTCGTTGGAGATGCTTGC	149	60.1/61.7	102.79	0.995
		Rev: CATTCCGTTGGGTCTAACTTCAG				
*MDH*	Malate dehydrogenase	For: ATCCAGTCAGATGCGTCGG	119	58.7/59.5	95.76	0.998
		Rev: GCTTTCTCTTCATACTCGGTCAAC				
*PEPC*	Phosphoenolpyruvate carboxykinase	For: TTTGGTGTCCTTCCCCCTG	106	60.2/60.8	94.66	0.999
		Rev: CCTTGATTCCATCCTCGGTTC				
*F-box*	F-box protein	For: AGTTCCACCGCTACCTGTCC	129	58.9/58.8	99.03	0.996
		Rev: ATTCGCCTTCAACGCCAA				
*OSBL*	o-Succinylbenzoate-CoA ligase	For: TGCTGGCTACACTGAGGATGA	143	58.9/60.3	95.27	0.999
		Rev: CCTTGACCGCTGCTTGAACT				
*ICS*	Isochorismate synthase	For: CATCCCTTCATCCAACTCCAG	218	59.2/58.5	98.46	0.998
		Rev: GCTTCCTTCTACCACGCCA				
*IPPI*	Isopentenyl-diphosphate delta-isomerase	For: AACCGAGACGAGTTGAGGGA	152	59.3/58.0	93.75	0.996
		Rev: ATGTCAATAGCATCAGTCAGGGT				
*HMGR*	3-Hydroxy-3-methylglutaryl-coenzyme A reductase	For: TTGAGGTCGGGACAGTAGGTG	116	59.8/58.6	93.79	0.998
		Rev: CAGCAGCCGAGCATTTGA				
*DXS*	1-Deoxy-D-xylulose-5-phosphate synthase	For: TTCTCTGCCTACGGCTACTCTT	119	58.2/58.8	94.68	0.996
		Rev: AACTTTTCGCTGCCTCGC				
*AACT*	Acetyl-CoA acetyltransferase	For: TTGAGGTCGGGACAGTAGGTG	249	60.0/59.4	96.41	0.994
		Rev: CAGCAGCCGAGCATTTGA				

^*∗*^bp, Tm, *E*, and *R*^2^ indicate base pair, melting temperature, PCR efficiency, and correlation coefficient, respectively. Standard curves of 15 candidate reference genes and six target genes are shown in Additional [Supplementary-material supplementary-material-1].

**Table 2:** Gene expression stability across sample sets calculated by NormFinder.

Rank	Total	PEG	NaCl	CuSO_4_	H_2_O_2_	MeJA	SA	Cold	Hot	Tissue
1	*TBP* (0.32)	*hnRNP* (0.03)	*EF-1α* (0.02)	*hnRNP* (0.04)	*hnRNP* (0.04)	*hnRNP* (0.07)	*eIF* (0.08)	*hnRNP* (0.02)	*RPL5* (0.03)	*hnRNP* (0.17)
2	*EF-1α* (0.34)	*eIF* (0.06)	*UBCE* (0.02)	*RPL5* (0.05)	*TBP* (0.05)	*RPL5* (0.09)	*hnRNP* (0.09)	*PTBP2* (0.03)	*hnRNP* (0.03)	*TBP* (0.21)
3	*UBCE* (0.42)	*RPL5* (0.06)	*hnRNP* (0.02)	*PEPC* (0.08)	*MDH* (0.07)	*TBP* (0.15)	*TBP* (0.10)	*F-box* (0.03)	*EF-1α* (0.05)	*MDH* (0.41)
4	*MDH* (0.49)	*MDH* (0.06)	*TUB* (0.04)	*EF-1α* (0.11)	*RPL5* (0.07)	*ACT2* (0.22)	*EF-1α* (0.11)	*RPL5* (0.04)	*TBP* (0.06)	*EF-1α* (0.41)
5	*RPL5* (0.51)	*UBCE* (0.13)	*TBP* (0.06)	*MDH* (0.12)	*eIF* (0.08)	*MDH* (0.29)	*UBCE* (0.12)	*PP2A* (0.09)	*UBCE* (0.09)	*RPL5* (0.44)
6	*hnRNP* (0.52)	*SAND* (0.13)	*RPL5* (0.11)	*TBP* (0.13)	*ACT2* (0.11)	*PTBP2* (0.30)	*ACT2* (0.15)	*EF-1α* (0.11)	*PP2A* (0.20)	*UBCE* (0.44)
7	*PTBP2* (0.58)	*ACT2* (0.13)	*MDH* (0.15)	*eIF* (0.15)	*TUB* (0.16)	*EF-1α* (0.30)	*PEPC* (0.17)	*MDH* (0.13)	*PTBP2* (0.22)	*F-box* (0.44)
8	*eIF* (0.58)	*PTBP2* (0.15)	*SAND* (0.18)	*ACT2* (0.17)	*UBCE* (0.16)	*SAND* (0.33)	*RPL5* (0.18)	*UBCE* (0.14)	*TUB* (0.29)	*PEPC* (0.47)
9	*PP2A* (0.60)	*TBP* (0.15)	*PEPC* (0.20)	*SAND* (0.19)	*F-box* (0.17)	*TUB* (0.37)	*SAND* (0.21)	*PEPC* (0.16)	*eIF* (0.32)	*ACT2* (0.50)
10	*PEPC* (0.63)	*PP2A* (0.16)	*PP2A* (0.21)	*PP2A* (0.24)	*PTBP2* (0.19)	*eIF* (0.44)	*PP2A* (0.22)	*TUB* (0.18)	*F-box* (0.33)	*PP2A* (0.52)
11	*TUB* (0.65)	*F-box* (0.18)	*PTBP2* (0.22)	*UBCE* (0.24)	*SAND* (0.20)	*UBCE* (0.55)	*PTBP2* (0.22)	*eIF* (0.20)	*SAND* (0.35)	*TUB* (0.66)
12	*ACT2* (0.67)	*GAPDH* (0.20)	*ACT2* (0.24)	*GAPDH* (0.24)	*EF-1α* (0.24)	*PEPC* (0.65)	*GAPDH* (0.30)	*TBP* (0.20)	*ACT2* (0.39)	*GAPDH* (0.66)
13	*SAND* (1.21)	*PEPC* (0.22)	*eIF* (0.38)	*PTBP2* (0.54)	*GAPDH* (0.24)	*GAPDH* (0.80)	*F-box* (0.31)	*ACT2* (0.28)	*GAPDH* (0.40)	*eIF* (0.75)
14	*GAPDH* (1.23)	*TUB* (0.23)	*GAPDH* (0.45)	*TUB* (0.63)	*PEPC* (0.26)	*PP2A* (0.81)	*MDH* (0.41)	*SAND* (0.28)	*MDH* (0.64)	*PTBP2* (0.84)
15	*F-box* (1.25)	*EF-1α* (0.23)	*F-box* (0.47)	*F-box* (0.64)	*PP2A* (0.29)	*F-box* (1.16)	*TUB* (0.42)	*GAPDH* (0.31)	*PEPC* (0.79)	*SAND* (2.08)

**Table 3:** Expression stability values for *R. yunnanensis* candidate reference genes calculated by BestKeeper.

Rank	Total	PEG	NaCl	CuSO_4_	H_2_O_2_	MeJA	SA	Cold	Hot	Tissue
1	*hnRNP*	*ACT2*	*F-box*	*F-box*	*PP2A*	*MDH*	*SAND*	*ACT2*	*F-box*	*UBCE*
	2.66 ± 0.65^*∗*^	0.23 ± 0.06	0.77 ± 0.18	0.74 ± 0.17	0.49 ± 0.11	1.50 ± 0.39	0.33 ± 0.09	1.07 ± 0.29	0.99 ± 0.23	2.35 ± 0.51
2	*TBP*	*RPL5*	*RPL5*	*SAND*	*PEPC*	*RPL5*	*TUB*	*TBP*	*eIF*	*PP2A*
	3.17 ± 0.73	0.45 ± 0.10	1.09 ± 0.24	1.50 ± 0.41	0.54 ± 0.11	1.88 ± 0.41	0.68 ± 0.18	1.52 ± 0.36	1.11 ± 0.25	2.87 ± 0.72
3	*PP2A*	*TUB*	*TUB*	*MDH*	*MDH*	*ACT2*	*hnRNP*	*UBCE*	*PEPC*	*GAPDH*
	3.24 ± 0.80	0.53 ± 0.14	1.19 ± 0.33	1.79 ± 0.46	0.75 ± 0.16	2.10 ± 0.56	0.75 ± 0.19	1.52 ± 0.37	1.42 ± 0.34	3.36 ± 0.80
4	*TUB*	*eIF*	*TBP*	*TBP*	*F-box*	*hnRNP*	*UBCE*	*PEPC*	*PTBP2*	*hnRNP*
	3.52 ± 0.97	0.74 ± 0.17	1.25 ± 0.29	1.98 ± 0.47	0.80 ± 0.14	2.23 ± 0.55	0.93 ± 0.21	1.76 ± 0.41	1.43 ± 0.34	3.60 ± 0.89
5	*RPL5*	*hnRNP*	*hnRNP*	*hnRNP*	*PTBP2*	*SAND*	*TBP*	*hnRNP*	*PP2A*	*PEPC*
	4.08 ± 0.89	0.75 ± 0.19	1.37 ± 0.33	2.39 ± 0.59	0.99 ± 0.23	2.45 ± 0.64	0.96 ± 0.22	1.94 ± 0.47	1.48 ± 0.36	3.67 ± 0.82
6	*SAND*	*F-box*	*MDH*	*ACT2*	*hnRNP*	*GAPDH*	*PP2A*	*PTBP2*	*TBP*	*TBP*
	4.55 ± 1.19	0.77 ± 1.18	1.44 ± 0.35	2.45 ± 0.63	1.06 ± 0.25	2.59 ± 0.69	1.01 ± 0.24	1.94 ± 0.51	2.14 ± 0.50	4.00 ± 0.92
7	*eIF*	*MDH*	*SAND*	*PP2A*	*ACT2*	*PTBP2*	*ACT2*	*F-box*	*TUB*	*MDH*
	4.58 ± 1.08	1.03 ± 0.25	1.46 ± 0.37	2.63 ± 0.65	1.09 ± 0.27	2.84 ± 0.73	1.46 ± 0.40	2.00 ± 0.47	2.36 ± 0.63	4.12 ± 0.99
8	*MDH*	*SAND*	*PEPC*	*eIF*	*TBP*	*eIF*	*eIF*	*TUB*	*hnRNP*	*ACT2*
	4.73 ± 1.16	1.05 ± 0.27	1.62 ± 0.33	2.69 ± 0.64	1.16 ± 0.26	2.94 ± 0.70	1.64 ± 0.40	2.02 ± 0.58	2.77 ± 0.67	44.94 ± 1.22
9	*UBCE*	*PTBP2*	*ACT2*	*GAPDH*	*TUB*	*TBP*	*PEPC*	*PP2A*	*RPL5*	*PTBP2*
	4.76 ± 1.06	1.08 ± 0.26	1.68 ± 0.42	2.73 ± 0.68	1.17 ± 0.32	3.11 ± 0.74	1.97 ± 0.44	2.03 ± 0.51	2.90 ± 0.65	5.15 ± 1.25
10	*PTBP2*	*UBCE*	*UBCE*	*PEPC*	*UBCE*	*TUB*	*F-box*	*RPL5*	*EF-1α*	*RPL5*
	5.16 ± 1.28	1.37 ± 0.29	1.73 ± 0.37	3.01 ± 0.66	1.33 ± 0.28	3.76 ± 1.06	2.05 ± 0.51	2.10 ± 0.48	2.94 ± 0.57	5.22 ± 1.17
11	*ACT2*	*PP2A*	*EF-1α*	*RPL5*	*RPL5*	*UBCE*	*PTBP2*	*MDH*	*UBCE*	*F-box*
	5.34 ± 1.37	1.38 ± 0.34	1.96 ± 0.36	3.06 ± 0.67	1.44 ± 0.28	4.02 ± 0.94	2.07 ± 0.54	2.48 ± 0.64	3.19 ± 0.75	5.59 ± 1.33
12	*PEPC*	*TBP*	*PP2A*	*EF-1α*	*eIF*	*EF-1α*	*EF-1α*	*EF-1α*	*SAND*	*TUB*
	5.45 ± 1.20	1.48 ± 0.33	2.05 ± 0.52	3.09 ± 0.60	1.50 ± 0.34	4.11 ± 0.83	2.08 ± 0.42	2.53 ± 0.51	3.67 ± 1.03	6.16 ± 1.69
13	*EF-1α*	*GAPDH*	*PTBP2*	*UBCE*	*SAND*	*F-box*	*GAPDH*	*SAND*	*ACT2*	*SAND*
	5.60 ± 1.09	1.84 ± 0.40	2.68 ± 0.65	3.25 ± 0.74	1.85 ± 0.46	4.11 ± 1.09	2.18 ± 0.60	2.89 ± 0.81	3.84 ± 1.07	7.03 ± 1.78
14	*F-box*	*EF-1α*	*eIF*	*TUB*	*GAPDH*	*PEPC*	*RPL5*	*eIF*	*GAPDH*	*EF-1α*
	6.22 ± 1.46	1.87 ± 0.35	3.61 ± 0.84	3.77 ± 1.05	1.94 ± 0.49	4.92 ± 1.10	2.37 ± 0.51	3.02 ± 0.72	4.00 ± 1.09	7.15 ± 1.41
15	*GAPDH*	*PEPC*	*GAPDH*	*PTBP2*	*EF-1α*	*PP2A*	*MDH*	*GAPDH*	*MDH*	*eIF*
	7.09 ± 1.75	1.96 ± 0.40	4.01 ± 0.89	4.46 ± 1.20	2.19 ± 0.39	5.04 ± 1.26	3.14 ± 0.79	3.25 ± 0.83	5.49 ± 1.38	7.53 ± 1.80

^*∗*^Fifteen candidate reference genes are evaluated by the lowest values of the coefficient of variance (CV) and standard deviation (SD), and these values are showed as CV ± SD.

**Table 4:** Comprehensive ranking of 15 candidate reference genes integrated by RefFinder.

Rank^*∗*^	Total	PEG	NaCl	CuSO_4_	H_2_O_2_	MeJA	SA	Cold	Hot	Tissue
1	*TBP (1.19)*	*hnRNP *(2.34)	*EF-1α *(1.68)	*hnRNP *(1.97)	*PTBP2 *(2.11)	*RPL5 *(1.68)	*TBP *(1.97)	*RPL5 *(2.14)	*RPL5 *(2.06)	*PTBP2 *(2.34)
2	*hnRNP *(3.08)	*MDH *(3.03)	*UBCE *(2.45)	*RPL5 *(2.58)	*hnRNP *(2.21)	*MDH *(1.86)	*hnRNP *(2.45)	*PTBP2 *(3.56)	*hnRNP *(2.34)	*hnRNP *(2.94)
3	*EF-1α *(3.56)	*eIF *(3.83)	*hnRNP *(3.57)	*PEPC *(3.08)	*TBP *(2.82)	*hnRNP *(2.43)	*UBCE *(2.99)	*hnRNP *(3.83)	*TBP *(3.94)	*TBP *(3.13)
4	*PP2A *(3.83)	*RPL5 *(3.87)	*TUB *(4.00)	*MDH* (4.40)	*MDH* (4.47)	*ACT2 *(3.46)	*eIF *(3.13)	*EF-1α *(3.98)	*EF-1α *(4.28)	*EF-1α *(4.12)
5	*RPL5 *(4.23)	*TBP *(4.43)	*TBP *(4.40)	*EF-1α *(4.43)	*RPL5 *(4.95)	*TBP *(5.42)	*SAND *(5.05)	*F-box *(4.05)	*UBCE *(5.36)	*UBCE *(4.23)
6	*UBCE *(4.82)	*EF-1α* (5.19)	*RPL5 *(4.74)	*TBP *(6.00)	*F-box *(6.45)	*PTBP2 *(5.89)	*EF-1α *(5.48)	*PP2A *(4.90)	*F-box *(5.61)	*MDH *(6.19)
7	*MDH *(6.45)	*ACT2* (6.15)	*MDH *(7.48)	*SAND *(6.34)	*PEPC *(6.84)	*EF-1α *(7.36)	*ACT2 *(6.24)	*UBCE *(6.05)	*PP2A *(5.73)	*F-box *(6.21)
8	*eIF *(6.96)	*SAND* (6.16)	*F-box *(7.62)	*eIF *(7.24)	*eIF *(7.14)	*SAND *(7.52)	*PEPC *(7.65)	*PEPC *(6.70)	*eIF *(5.83)	*PP2A *(6.67)
9	*TUB *(7.88)	*UBCE* (7.33)	*PEPC *(7.98)	*F-box *(7.62)	*ACT2 *(7.24)	*eIF *(7.84)	*PP2A *(8.57)	*ACT2 *(6.71)	*PTBP2 *(6.09)	*PEPC *(6.93)
10	*PTBP2 *(9.08)	*PTBP2* (8.74)	*PP2A *(8.21)	*ACT2 *(8.38)	*PP2A *(9.06)	*TUB *(9.93)	*TUB *(9.06)	*TBP *(6.83)	*TUB *(8.71)	*RPL5 *(6.96)
11	*PEPC *(10.70)	*TUB *(10.00)	*SAND *(9.46)	*PP2A *(8.57)	*TUB *(9.19)	*GAPDH *(10.70)	*RPL5 *(9.16)	*MDH *(9.32)	*PEPC *(10.00)	*GAPDH *(9.00)
12	*SAND *(12.20)	*F-box *(10.60)	*ACT2 *(11.00)	*GAPDH *(10.60)	*UBCE *(9.74)	*UBCE *(11.20)	*PTBP2 *(11.50)	*TUB *(10.50)	*SAND *(11.00)	*ACT2 *(10.20)
13	*ACT2 *(12.20)	*PP2A *(10.70)	*PTBP2 *(12.20)	*UBCE *(12.00)	*SAND *(11.70)	*PEPC *(12.20)	*F-box *(12.50)	*eIF *(12.50)	*GAPDH *(12.70)	*TUB *(12.50)
14	*GAPDH *(14.20)	*GAPDH *(12.20)	*eIF *(13.20)	*PTBP2 *(13.50)	*EF-1α *(12.50)	*PP2A *(14.20)	*GAPDH *(12.70)	*SAND *(14.00)	*ACT2 *(12.70)	*eIF *(14.00)
15	*F-box *(14.70)	*PEPC *(13.50)	*GAPDH *(14.20)	*TUB *(14.00)	*GAPDH *(14.00)	*F-box *(14.50)	*MDH *(14.20)	*GAPDH *(15.00)	*MDH *(14.20)	*SAND *(14.70)

^*∗*^RefFinder exploits computational programs (such as BestKeeper, geNorm, Normfinder, or the comparative delta-ct method) to rank and compare candidate reference genes. The values following the genes indicate the geometric mean of the attributed weights measured by this software for the overall final ranking.

**Table 5:** Six putative key genes involved in the biosynthetic pathways of anthraquinones in *R. yunnanensis*.

Enzyme name	Abbreviation	EC number	Query ID
Shikimate pathway key genes forming rings A and B			
*Isochorismate synthase*	*ICS*	5.4.99.6	TRINITY_DN125672_c2_g2
*o-Succinylbenzoate-CoA ligase*	*OSBL*	6.2.1.26	TRINITY_DN125216_c2_g1
*Isopentenyl-diphosphate isomerase*	*IPPI*	5.3.3.2	TRINITY_DN131965_c2_g1

Terpenoid pathway key genes forming ring C			
*Acetyl-CoA acetyltransferase*	*AACT*	2.3.1.9	TRINITY_DN130086_c0_g2
*3-Hydroxy-3-methylglutaryl-coenzyme A reductase*	*HMGR*	1.1.1.34	TRINITY_DN129754_c4_g1
*1-Deoxy-D-xylulose 5-phosphate synthase*	*DXS*	2.2.1.7	TRINITY_DN124410_c3_g1

## Data Availability

The data used to support the findings of this study are included within the article.

## Supplementary Materials

Supplementary Materials Additional Figure S1: standard curves of 15 candidate reference genes and six target genes were directly generated by StepOne™ Real-time PCR system. Additional File 1: sequence information of 15 candidate reference genes and six target genes selected from *R. yunnanensis* transcriptome database. Table S1: information of 15 candidate reference genes and six target genes selected for evaluation and validation based on *R. yunnanensis* transcriptome database. Table S2: raw Ct values in *R. yunnanensis.* Table S3: expression stability ranking of the 15 candidate reference genes estimated by geNorm, NormFinder, BestKeeper, and RefFinder.
