# Supplementary material for: Selection and Validation of Appropriate Reference Genes for Quantitative RT-PCR Analysis in Rubia yunnanensis Diels Based on Transcriptome Data
Source: Biomed Res Int. 2020 Jan 8;2020:5824841. doi: 10.1155/2020/5824841 (PMC6973195; doi:10.1155/2020/5824841)
Supplement: Supplementary Materials — Additional Figure S1: standard curves of 15 candidate reference genes and six target genes were directly generated by StepOne™ Real-time PCR system. Additional File 1: sequence information of 15 candidate reference genes and six target genes selected from R. yunnanensis transcriptome database. Table S1: information of 15 candidate reference genes and six target genes selected for evaluation and validation based on R. yunnanensis transcriptome database. Table S2: raw Ct values in R. yunnanensis. Table S3: expression stability ranking of the 15 candidate reference genes estimated by geNorm, NormFinder, BestKeeper, and RefFinder. [file 5824841.f1.zip › 5824841.f1/Additional Figure S1.pdf]

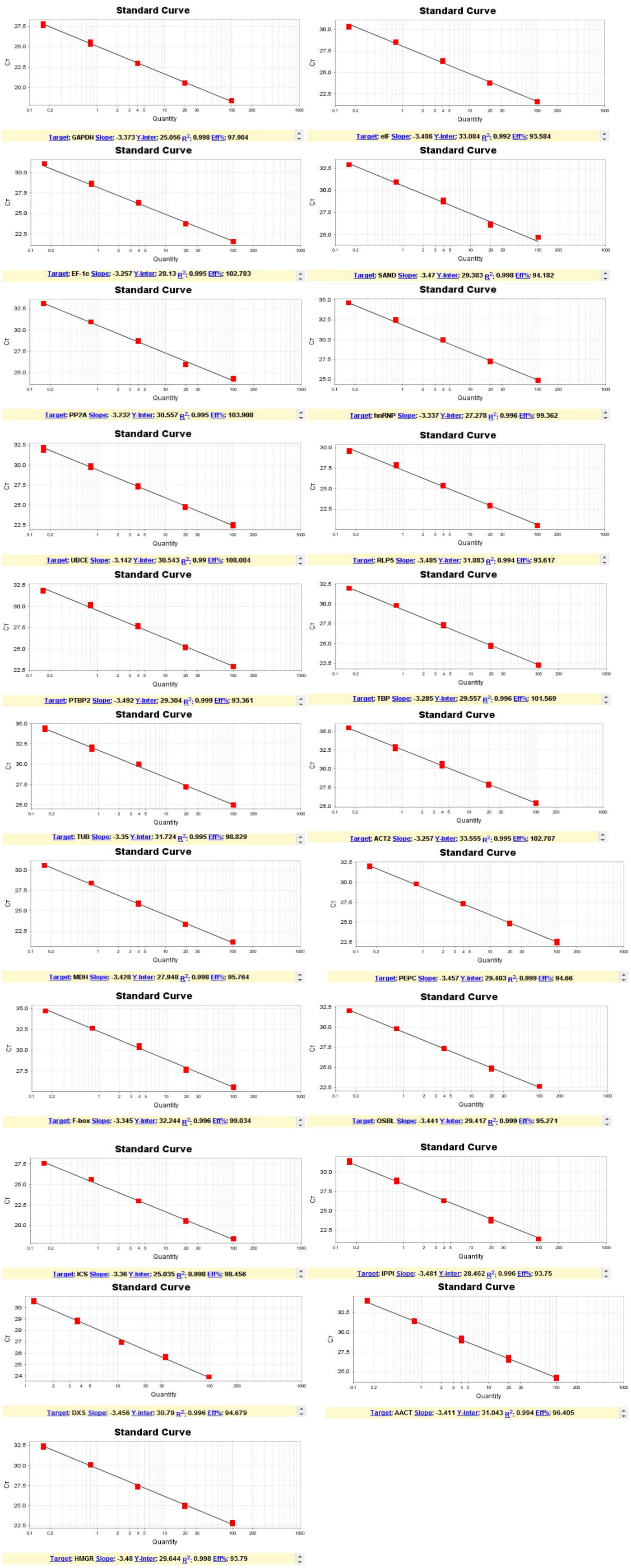

Supplemental fig. 1 Standard curves of 15 candidate reference genes and six target genes were directly generated by StepOne^{TM} Real-time PCR system.
