# Supplementary material for: Selection and Validation of Appropriate Reference Genes for Quantitative RT-PCR Analysis in Rubia yunnanensis Diels Based on Transcriptome Data
Source: Biomed Res Int. 2020 Jan 8;2020:5824841. doi: 10.1155/2020/5824841 (PMC6973195; doi:10.1155/2020/5824841)
Supplement: Supplementary Materials — Additional Figure S1: standard curves of 15 candidate reference genes and six target genes were directly generated by StepOne™ Real-time PCR system. Additional File 1: sequence information of 15 candidate reference genes and six target genes selected from R. yunnanensis transcriptome database. Table S1: information of 15 candidate reference genes and six target genes selected for evaluation and validation based on R. yunnanensis transcriptome database. Table S2: raw Ct values in R. yunnanensis. Table S3: expression stability ranking of the 15 candidate reference genes estimated by geNorm, NormFinder, BestKeeper, and RefFinder. [file 5824841.f1.zip › 5824841.f1/Additional file 1.doc]

**Sequence information of 15 candidate reference genes and six target genes selected from *R. yunnanensis* transcriptome database**

1. glyceraldehyde-3-phosphate dehydrogenase (*GAPDH*)

GCTGGGAGTGGAGATTTTGCTGAGATTCTTGATGGGGAGGTTTTTAAGTACTATGCTGATGGAGAGTGGAAAAAGTGTGGTTCTGGGAAATCTGTTGCCATAGTAAATCCTACCACAAGAAAAACCCAGTACAAGGTTCAAGCATGTTCACAAGAAGAGGTTAATAAGGTGATGGAGAGCTCGAAAGCTGCGCAAAAACTGTGGGCGAGAACGCCTCTGTGGAAGCGCGCTGAGCTGCTTCACAAGGCAGCTGGCATTCTTAAAGAACACAAAGCCCCAATTGCAGAATGCCTTGTCAAAGAAATCGCAAAACCGGCAAAAGATGCTGTAACTGAGGTTGTAAGATCTGGAGATCTGGTTTCTTATTGCGCGGAAGAAGGAGTGAGGATATTGGGGCAAGGGGAATTCTTAGTTTCTGATAGTTTTCCTGGTAATGAGAGGACTAAATACTGCCTCACTTCCAAGATTCCGCTTGGTGTTGTTTTAGCTATCCCTCCGTTCAACTATCCCGTCAACCTCGCTGTCTCCAAAATTGGACCAGCCTTGATTGCTGGAAATTCCATAGTGCTGAAGCCTCCAACTCAGGGTGCTGTTGCTGCCCTTCATATGGTGCACTGTTTCCACTTAGCCGGTTTCCCGAAAGGCCTCATAAGCTGTGTTACCGGAAAAGGTTCTGAAATAGGCGACTTTTTAACCATGCATCCTGGTGTCAACTGTATCAGCTTCACGGGTGGTGACACTGGTGTTGCGATCTCCAAAAAGGCGGGAATGGTTCCGCTGCAAATGGAGCTCGGAGGAAAAGATGCCTGCATTGTTCTTGAGGATGCAGATTTGGATTTGGTTGCAGCAAACATCATCAAAGGAGGCTTCTCTTACAGCGGCCAGAGGTGCACCGCTGTTAAGGTTGTCCTGGTGATGGAATCTGTTGCTGACGATCTTGTTGAAAAGGTTAAGGCCAAGGTGGCTAAACTGAAAGTTGGACCTCCTGAGGACGACTGTGACATCACCCCGGTTGTATCTGAGTCCTCAGCAAACTTCATTGAAGGGCTAGTCACCGACGCTAAAGAGAAAGGTGCAACATTTTGCCAGGAGTATAAGAGGGAGGGCAATCTCATCTGGCCACTGTTGTTGGATCACGTCAGGCCGGACATGAGGATCGCTTGGGAGGAGCCGTTCGGGCCTGTTTTACCCGTCATTAGAATAACCTCTGTTGAAGAAGGGATTCACCATTGCAATGCCAGCAATTTTGGCCTCCAGGGATGTGTTTTCACCAAAGATATCAACAAGGCCATACTGATCAGTGATGCAATGGAGACAGGAACGGTCCAGATCAACTCGGCTCCAGCTCGTGGGCCGGACCATTTCCCGTTCCAGGGAATCAAGGACAGCGGGATTGGATCACAGGGCATCACGAACAGCATC

2. eukaryotic translation initiation factor 3 subunit D-like, partial (*eIF*)

GACCGCCTCTACAACCTCAACCGCTCGACGACGAACTCCGGTCCTCGCCGCGAGTCCTCCGTCTTCAAATCCTCTGTTGATATCCAGCCCGAGTGGAATATGCTCGACCAGATCCCGTTCTCCACATTCTCGAAGCTCTCTTTCTCTGTCCCGGAGCCTGAAGACCTTCTCATCTGTGGCGGGCTCGAGTTCTACGACCGCGCCTACGACCGAGTCACTCCCAGGAATGAACGCCGTTTGGAGAGGTTCAAGAACCGTAACTTCTTTAAGATAACCACTACAGACGACCCCGTGATTCGCCGACTTGCCAATGAGGATAAGGCCACGGTTTTTGCGACGGACACCATTCTGTCAACGCTTATGTGCGCTCCGAGGTCAGTTTACTCGTGGGACATCGTGATCCAGCGCGTTGGAAACAAGCTCTTCTTTGACAAGCGTGATGGGTCTCAGCTAGATTTGTTATCTGTCCATGAAACCTCTCAAGAGCCCTTGCCTGATGTCAAGGATGATATAAACTCTGCGTACTCTCTTAGTGTCGAGGCTGCGTATGTGAACCAGAACTTCTCGCAGCAGGTTCTGCATAGGGATGGTAACAAGGTGAGTTTTGAGGAGCCCAACCCTTTTGCGCCCGAAGGAGAAGAAGTGGCTTCTGTGGCTTATAGGTACAGGAGGTGGAAGCTTGATGATGATATGTATCTGGTTGCACGGTGTGAGGTGCAGAGTGTGGTGGATTTGAATAACCAGAGGTCGTTTTTGACACTGAATGCATTGAATGAGTTTGATCCCAAGTATTCTGGTGTGGATTGGAGGCAGAAGCTGGAGACTCAGCGTGGTGCTGTGCTGGCTACTGAGCTGAAGAACAATGCAAACAAGTTGGCAAAATGGACTGCACAGTCGCTGTTGGCTAGTGCTGATATGATGAAGCTGGGTTATGTTTCAAGAGTGCATCCTAGGGATCACTATAACCATGTCATTCTCGCTGTTGTTGGTTACAAGCCAAGGGATTTTGCTGGGCAGATTAATTTGAACACTTCTAATATGTGGGGTATTGTGAAGAGTATTGTGGACCTGTGTATGAAGTTGAACGAGGGCAAGTATGTTCTTGTTAAGGACCCCACGAAGCCGCAAGTGAGGATCTACGAGGTTCCGCCTGATGCGTTTGAGAACGATTATGTGGAGGAGCCTTTGCCAGAGGATGAACAGGTTCAACCACCTGCTGAGGATGCTGAAGCTGTGGATGGTATCGCAGAAGCGAACAATGTGGAGGACAAAGAG

3. elongation factor 1-alpha (*EF-1α*)

TGGGTGCTTGACAAGCTCAAGGCTGAGCGTGAGAGAGGTATCACCATTGATATCGCTCTGTGGAAGTTTGAGACCACCAAGTACTACTGCACAGTCATTGATGCCCCTGGACATCGTGATTTCATCAAGAACATGATCACGGGTACCTCACAGGCTGACTGTGCTGTTCTCATCATTGACTCCACTACTGGAGGTTTTGAGGCTGGCATTTCCAAGGATGGACAGACCAGGGAGCATGCTCTTCTTGCTTTCACACTTGGTGTTAAGCAGATGATCTGTTGTTGTAACAAGATGGATGCCACCACCCCCAAGTACTCCAAGGCTAGGTATGATGAAATCGTGAAGGAAGTTTCCTCCTACCTCAAGAAGGTTGGTTACAACCCAGACAAGATCAACTTCGTCCCCATCTCTGGGTTTGAAGGTGACAACATGATTGAGAGGTCCACCAACCTTGACTGGTACAAGGGACCAACCCTTCTTGAGGCCCTTGACATGATCAACGAGCCCAAGAGACCCTCAGACAAGCCACTCCGTCTTCCCCTCCAGGATGTGTACAAGATTGGTGGTATTGGAACTGTGCCAGTGGGTCGTGTTGAGACTGGTATCATCAAGCCTGGTATGGTAGTCACATTTGGCCCAACTGGGTTAACAACTGAGGTCAAGTCTGTGGAGATGCACCACGAGGCACTCCAGGAGGCTCTTCCTGGTGACAATGTTGGGTTCAATGTTAAGAATGTTGCTGTCAAGGATCTCAAGCGTGGGTTTGTGGCCTCCAACTCCAAGGATG

4. SAND family protein, partial (*SAND*)

ACAATTTCTAGGAATCCAGCTACCTTTCTTCATGCATATACATGCCTTCCCCTTGCGTATGCCACAAGACAAGCTGCTGGTGCAATCTTACAGGATGTTGCTGATTCAGGTGTCTTGTTTGCAATTTTAATGTGTAGACACAAGGTCCTTATAGCCGTCTCTAAGATTGCTTTAATTGTAAAGTTAGTTGTATATCCTATTTCACTGTTTGAATACTATGATTGGAAATTAATGCAGGTTATCTCTCTTGTTGGTGCACAAAAAGCATCCCTACATCCTGATGATATGCTTCTTTTGTCCAACTTTGTTGTGTCATCAGAATCGTTCAGGACATCTGAATCTTTCTCACCTATTTGCCTTCCAAGATACAATCCAATGGCGTTTCTGTATGCATATGTCCATTATTTTGATCTTGACACATACTTGATATTGCTCACCACAAGTTCAGATGCATTTTACCATCTAAAAGATTGCAGGATTCGTGTTGAGAATGTACTTCTGAAGTCAAATGTGCTTGTTGAAGTTCAGAAGTCTTTGTTGGAAGGTGGCATGCATGTTGAGGATTTGTCTATTGACTCTACTTCTCGTTCTGCAACTGTTTCACCTCATGCTAGTCAGCTACGGCTTGCAACTGAATCCACAGAGCGATTAAGGGACACTTTCAGTACTGTTGGTGGTCCTGCTGGACTCTGGCATTTTATATACCGGAGTATTTATCTCGACCAATATGTAACATCTGAATTTTCATCGCCAATTAATAGCACGCAACAACAAAAAAGGTTATTAAGAGCTTATCAGAAGATGTATGCATCCATGCATGATAAAGAAATTGGACCATACAAAACTCAATTCAGAAGGGATGAGGATTATGTTCTGCTTTGCTGGGTTACTCAAGATTTTGAACTCTATGCTGCATTTGATCCCCT

5. serine/threonine-protein phosphatase 2A activator isoform X2 (*PP2A*)

TCTAATTTGGTATTTACAGGTACGGGCCATGAAACAAATTTTGCAGCGTGGCTATATTGC

TTAGCTAGATTGCAGATTGTGAAAGAAGAGGATTATCCAGCCTTGGTGTCAAGGGTATTCACAAAGTACTTGGATTTGATGCGAAAATTGCAGCTAACATATTCCTTAGAGCCAGCTGGATCTCACGGAGTTTGGGGACTTGACGATTACCACTTTTTGCCTTTCATCTTTGGTTCTTCGCAGTTGATTGACCACAAGTATATGAAGCCAAAGTCGATTCATAACGAGGATATATTGGAGAATTTTACAAGCGAGTATCTGTATCTCTCTTGTATTGTATTTATAAAGAAGGTTAAAAAGGGACTCTTTGCAGAACATTCTCCAATGTTGGATGATATCAGTGGCGTGCCAAATTGGAACAAGGTCAATAGCGGGCTCTTGAAGATGTACAAAGTGGAAGTGTTGCAGAAGGTCCCTATCATGCAACATTTCCTATTTGGTTCGATAATCAAATGGGAATGATCTGAGATGCTCTTCATTGCACAGTGAGGAGGTTCTGGAGTTGCAGCTTTATAGATCTATGATTTTGTCTTTATTTAGTTGTAATACATGTTGTGACATTAATTAGTTAGTGAAAAATTGATGACAATTTTATCAGTTTCATCGCACGTGTTGGCTTGATGTTTACCACAGATGTTCACTGCTACCTGAAGTTTGGAATTCTTCTGGGTGTTTGTACTGCAACTCATGCATACCAGATGCTCATTATTATCTAGACAAATACCAGAGGTTAGATTGGTGATAAAGAACATTTTGGTGTATGAATATCAATGTGAAATCTCGTACACATAGCTCTTGATGAGCAGATGCTACCAAATCAGCAATGCGGTTGGTAAGCAGCATTTTGTGAAGTTGAGGTTTAGGAGAAAACTGAATTTGCTGACGAAGGCTAAAAAATGACATGTCATACAGAG

6. heterogeneous nuclear ribonucleoprotein A1-like 2 (*hnRNP*)

ATGTCCGACAGAAAGCTTGTAGTCTTAGGTATCCCGTGGGATGTGGAAACCGAAGGTTTGAGGGAATACATGGCCAAGTTTGGGGATTTGGAGGACTGCATTGTCATGAAGGAGAGGTCTACTGGACGATCTAGGGGATTCGGATATGTGACTTTTGCGACAGCTGGAGATGCAAAGGCAGCACTGTCAAGTGAGCATACACTTGGTAATAGAATACTGGAAGTCAAAATTGCCACTCCAAAGGAGGAAATGAGGTCATCATCCAAAAAAATCACCAGGATTTTTGTTGCACGGATTCCCCCAGCTGTGTCTGAAGCAGCATTCCGAAGTTATTTTGAGAAATACGGGAACATTACCGATCTTTACATGCCCAAGGATCCAAGTACTAAAGCACATCGTGGAATTGGGTTCGTCACTTTTGTTAATTCGGAATCTGTGGATGATCTAATGGCCGAGACCCATGAGTTGGGGGGCTCTACAATTGTTGTCGATCGAGCCACTCCCAAGGAGGATGAGATCAGGCCAGTAAGCCGAATGCCTCCAAGTGGTTATGGTGCATATAATTCTTACATGAATTCTGCAACCAGATATGCAGCATTAGGTGCTCCCACCTTATACGACCATCCTGGCTCTATGTATGGAGGAAGAAATGGCCCAGTAGCCCCTCCACGTGGTATGGGCAAAAAGATTTTTGTTGGGCGTCTCCCCCAGGAGGCTAGTGCTGAAGATCTTCGACAGTATTTCGGTAGATTTGGCCGGATTTTTGATGTATATGTCCCAAAGGATCCAAAGAGAACTGGTCATAGGGGATTTGGTTTTGTCACCTTTGCTGATGATGGTGTTGCAGATCGTGTGTCACGTAGATCTCATGAGATTTTTGGGCAACAGGTTGCAATTGATTCAGCCACGCCACTTGATGATGCTGGTCCAAGTAGTAGTTTTATGATGGATAACCCTGAACCTTATGGTGGTTATGGTGGTGGTCCTATGCGTCCTTATGGTAGAATGCATGGTGGGATGGAGTTTGATGATTGGGGCTATGGGATGGGCTCCGGGAGGCCATCCAGAGCAGATTTTAGGTATAGGCCTTATTAGACGTGGAAAGATACAATTGATGCCTTATCTAAGATTGACGACTTGTGAAATACAATATCTTCTTTAGTCGAGTTATAAAGTGTAT

7. ubiquitin-conjugating enzyme E2 4 (*UBCE*)

ATGTCTTCCCCCAGCAAACGCCGAGAGATGGATTTAATGAAACTGATGATGAGTGACTATAAGGTGGATATGGCCAATGATGGCATGCAGGAATTTACAGTGGAATTTCACGGCCCTAAGGAAAGTCCTTATCAGGGAGGTGTGTGGTGCATACGAGTGGAGTTGCCGGATGCTTATCCGTACAAATCTCCGTCGATTGGTTTTGTGAACAAGATATACCATCCCAATGTTGACGAAATGTCAGGTTCAGTTTGTTTGGATGTTATCAATCAGACATGGAGCCCGATGTTTGATCTGGTTAATGTATTTGAGGTGTTCCTTCCTCAACTTCTGCTGTACCCCAATCCGTCTGACCCTTTGAATGGGGAAGCTGCTGCTCTCATGATGCGTGATCGTGCTGCATATGATCAAAGAGTAAAAGAGTACTGTGAAAAATATGCCAAGCCTGAGGATGCCGGGGCCGTGCCAGAGGAGAAATCTAGTGATGACGAGGTTAGCCAAGATGAATACGCCTCTAGTGACGAGGAAGTTGCTGGAAAAGCCGATCCCTGAGTGGATATAAAAATTTCTTTAACCGTCTGTATGTACATGAATAAGTTCTATATATAACTCAGTACCGTTTCCTGAATGAATTGTTCATGCTTCTGTTTCCGTATCCCACGGTAATTGTTCATATTGATTCATTCTGGCTTAGGACAAGTTACTTATGATGATTTAATTGTGCTTTTGTGTGGTGAAAAGAGTGGTGCTGCTTAGGATGAATGCCTGGAATCAATGCTTGATTTTGTTAATGGAGATTGTGCTTCTTTACAAAATCAAGCATTGATTCC

8. 60S ribosomal protein L5 (*RPL5*)

ATGGTGTTCGTGAAATCCCAGAAGTCAAGGTCCTACTTCAAGAGGTACCAGGTCAAATTCAAGAGAAGGAGAGAGGGAAAGACTGACTATCGTGCTAGGGTCCGATTGATCAATCAGGACAAGAACAAGTATAATACGCCTAAGTACCGGTTTGTTGTGAGGTTCACCAACAAGGACATTGTTGCACAGGTTGTCTCTGCTAGCATCACTGGTGACCATGTTCTTGCTGCTGCTTATGCGCATGAGTTGCCCCGTTATGGTCTTAAACTTGGCCTAACTAACTATGCTGCTGCCTACTGCACTGGTCTGTTACTTGCCCGCCGTGTTCTCAAGAAGCTGGTAATGGATGCTGAGTATGAAGGGAATGTTGAGGCTACTGGTGAGGATTATTCTGTTGAACCATCTGAAAGCAGAAGGCCTTTCCGTGCTCTCTTGGATGTGGGCCTTATCAAAACCACTACTGGCAACAGAGTTTTTGGTGCACTGAAGGGTGCATTGGATGGTGGGCTTGACATTCCTCATAGTGAAAAGAGGTTTGCGGGGTTTGGTAAGGACTCCAAACAGCTTGATGCTGATGTTCACCACAAGTACATCTATGGGGGCCATGTCTCTGCATATATGAGAACACTGGAAGAAGATGAGCCAGAGCGGTACCAGTCTCACTTTAGCCAGTACCTTAAGAGTGGTGTTACAGCTGATGACATCGAGGCATTGTACAAGAAAGTCCACACAGCTATTCGTGCGGATCCAACTGAAAAGAAATCAGCTAAGAAGCCCGTGACTCAGCATAAAAGGTACAATCTTAAGAAACTGACTTATGAGCAAAGGAGGACCAAGTTAATCGAGAGGTTGAATGCATTGAATTCCGCTGGT

9. polypyrimidine tract-binding protein homolog 2 isoform X4 (*PTBP2*)

ATGGCATCAGTATCCAGTCAGCCTCAGTTCCGCTACACGCAGCCACCGTCTAAGGTTCTGCACTTGAGAAACTTACCGTGGGAATGTACTGAGGAGGAACTTGTCGAATTAGGGAAGCCCTTCGGTAGAGTTGTCAATACCAAGTGTAATGTTGGAGCAAATCGTAACCAAGCATTTATAGAGTTTGCGGAATTGAATCAGGCAATCGCGATGATATCTTACTATGCTTCATCATCCGAACCAGCTCAGGTGCGAGGGAAAACTGTCTATCTACAGTATTCCAACAGGCAAGAAATAGTGAACAACAAAACTACTGCAGACGTAGCTGGGAATGTGCTTCTGGTAACCATTGAGGGTAATGACGCTCGACTCGTTAGCATTGATGTTCTTCACTTGGTATTTTCTGCTTTTGGATTTGTGCATAAGATCACTACATTTGAAAAGACAGCAGGATTTCAAGCTCTAGTCCAATTTTCAGATGCGGAGACTGCTTCTTCGGCAAAGGATGCTCTTGATGGAAGA

10. TATA binding protein (*TBP*)

CTAGACTGCAAATTGGACCTGAAGTCCATTGCTTTGCAAGCTAGAAATGCAGAATATAATCCAAAGCGTTTTGCTGCTGTCATTATGAGAATTAGAGAGCCAAAAACTACGGCCTTAATTTTTGCCTCTGGAAAGATGGTTTGTACCGGAGCCAAGAGTGAGCAGCAGTCTAAATTGGCAGCTCGGAAGTATGCTAGAATCATCCAAAAGCTTAATTTTCCTGCCAAATTCAAGGATTTTAAAATACAGAACATTGTGGGTTCTTGTGATGTTAAATTCCCCATTAGATTGGAAGGGCTTGCTTACTCTCACGGAGCCTTTTCAAGCTATGAACCTGAACTCTTTCCTGGATTGATATATCGAATGAAACAACCGAAAATTGTACTGCTTATCTTCGTTTCTGGAAAAATTGTCCTTACTGGAGCAAAGGTTAGGGAGGAGACCTATGCTGCATTTGAGCTTATATACCCTGTTCTGACTGAGTTCAGGAAGAATCAACAATGATTGAGAGAGATGATTGACCAACTTTGTGGATGACAGAACCAGTCTTCATGTCTATGTGGGACGGCGGTGTATATTAGAGAGGGACTGGACTGGCAAGTCTGGCATGGCTATTGTTTGGGTTCTTTTAGATAATATAATGGAAGCAGCTGGTTCTACGGTGGAAAAATTAGTAGGGGCAACTAAAATTTTGTTTCTTGCTATGCTTTTAAGTTAGGCTTTCGATGGACCCCGAATTTGCTGGTCTTGTCTTGAATGTAATCTGAACGGAAACATATGTGCTTAATCTGAATTTTACATTCTCAGCCTAACTGTAATGCTCTAC

11. tubulin gamma-1 chain (*TUB*)

ATGCCGAGGGAAATCATCACTCTTCAAGTAGGTCAATGTGGGAATCAGATCGGCATGGAATTCTGGAAACAACTGTGTCTTGAGCATGGCATAAGCAAAGACGGCATTCTCGAAGACTTCGCTACTCAGGGAGGGGACAGGAAAGACGTGTTCTTCTATCAAGCTGATGATCAGCACTATATACCACGAGCCTTGTTGATGGACTTGGAGCCCAGGGTGATTAATGGTATTCAAAATGGTGAATACAGGAATCTATACAATCATGAGAATGTTTTTATAGCTGATCATGGAGGAGGTGCTGGGAATAACTGGGCGAGCGGGTATCATCAGGGTAAGCAATATGAGGAGGATTTAATGGATATGATCGATAGGGAAGCTGATGGAAGTGATAGCCTGGAGGGCTTTGTTCTTTGCCATTCAATTGCTGGAGGAACAGGATCAGGCATGGGTTCCTATCTACTGGAAACCTTAAATGATCGCTACAGCAAGAAACTGATTCAAACATACAGTGTATTTCCTAACCAGAATGAGACAAGTGATGTGGTGGTGCAGCCGTACAACTCCCTTTTGACACTCAAGAGACTTACACTGAATGCTGATTGTGTTGTTGTTCTTGATAATACTGCACTGAATAGAATTGCTGTGGAGCGGCTTCATATCTCGACTCCAACATTTGCACAAACAAACTCTTTAGTTTCTACTGTAATGTCAGCAAGCACAACAACATTGCGTTATCCTGGGTACATGAACAATGACTTAGTTGGCCTTCTTGCTTCTCTGATCCCAACTCCAAGATGCCACTTCCTGATGACCGGATATACACCACTAACTGTTGAACGACAGGCAAATGTAATTCGCAAAACAACTGTACTTGATGTGATGAGAAGACTTCTTCAGACAAAGAACATTATGGTTTCCTCTCATGCTAGAACAAAAGAGGCTAGTCAGGCAAAGTACATATCTATTCTAAATATTATACAGGGTGAAGTTGATCCCACTCAGGTTCATGAAAGTTTGCAAAGAATTCGCGAACGGAAACTAGTTAACTTTATTGAATGGGGACCTGCAAGCATCCAGGTTGCTCTCTCAAGAAAATCCCCCTATGTTCAGACTGCTCACAGGGTCAGCGGCTTGATGTTGGCCAGTCATACTAGTATCCGCCACTTATTCAGCAAGTGCCTAAGCCAGTATGACAAATTGAGGAAGAGACAAGCCTTTCTTGACAACTACAGAAGTCATCCAATGTTTGCTGACAATGATCTTTCAGAATTTGATGAATCTAGAGATATAATTGAGAGTCTTGTTGATGAGTACAAAGCTTGTGAATCCCCAGATTACATCAAGTGGGGGATGGAGGATCCTGACCACGTTCTTACTGGGGAAGGAAATGCTACTGGAACTGTGGACCCAAAACTAGCAGTT

12. actin-related protein 2 (*ACT2*)

GAGAATTTTCCAACATCTGTTTTTCCTTGCGTTGTGGGAAGGCCGATGCTGAGATATGAAGAATCCCTTATGGAGCAAGAAATCAAGGACATTGTCGTTGGAGATGCTTGCTTGAAATTGAGGCATCAATTGGATATTTCTTACCCTGTCAACAATGGGATAGTGCAAAATTGGGATGATATGGAACACATATGGGACCATGCATTTTATAATGAGCTGAAGTTAGACCCAACGGAATGTAAGATCTTGCTGACTGATCCGCCTCTGAATCCAGCAAAAAATCGTGAACTGATGGTTGAGACTATGTTTGAGAAGTATAACTTTGCCGGTGTCTTTATTCAGATTCAAGCAGTTCTAACATTGTATGCTCAAGGGTTGCTTACAGGATTAGTAATTGACTCTGGTGATGGTGTTACTCACGTGGTA

13. malate dehydrogenase, chloroplastic (*MDH*)

ATGGCAGCTACTCCAGCAACCACCCTTTCAATTGGATCAACTGCTTACCTTGGCCCCAAAAGCGGCTCAGTTTCTAACTCAAAGGCGTTTGGTGTTAGAATCAACTCCTCAAACTGTCTTAAGAGTTTCAGTGGCCTCAAGGCAGCCTCTTCCGTCAGCTGTGAACCAGATTCCTCATTTTTCGGCAAGGAAAGTTGTGCAGCCATTCGACAATCTTTTGTCTCAAAAGCTCAGAAGCAGGACGTAAAATCTTGGAACTATGTTCAACCTCAGGCATCTTACAAGGTGGCCGTCCTGGGGGCTGCTGGGGGTATAGGTCAGCCACTTGCGCTTTTGATCAAGATGTCGCCACTAGTATCAGCTCTTAATCTTTATGATATTGCGAACGTCAAGGGAGTTGCTGCGGATCTCAGCCACTGCAACACTCCATCCCAAGTGTCTGATTTCACAGGAGACCAGGAACTGGCCAGCTGCTTGAAAGATGTAAATGTCGTGGTCATACCTGCTGGCGTTCCTAGAAAGCCCGGAATGACCCGTGATGACTTGTTCAATATCAATGCCAGTATCGTGAAGAACTTAGTTGAGGCTGTTGCTGATAATTGCCCTGATGCCTTCATTCACATCATCAGTAATCCTGTCAATTCTACCGTGCCAATTGCAGCTGAGGTGCTTAAGAAGAAAGGAGTGTACAACCCCAAGAAACTCTTTGGGGTTACCACCCTTGATGTTGTCAGGGCCAACACATTCGTCGCACAGAAGAAAAAGCTGAGGCTGATTGATGTTGATGTCCCTGTTATTGGAGGACATGCAGGGATTACCATTCTCCCGCTCTTGTCAAAGACAAAGCCCTCCGTCACCTTCACAGATGAAGAAGTGCACGAATTGACTGTGAGGATCCAGAATGCAGGAACCGAGGTGGTTGAGGCAAAAGCTGGTGCAGGGTCTGCCACGCTTTCCATGGCTTATGCGGCAGCAAGATTTGTCGAGTCCTCCCTCCGCGCTCTTGATGGCGACAGTGACGTGTACGAGTGCTCTTACATCCAGTCAGATGCGTCGGATCTTCCATTCTTTGCATCAAGGATTAAGCTTGGAAAAGATGGTGTTGAGGCTGTGATCAAGTCTGACCTGCAGGGGTTGACCGAGTATGAAGAGAAAGCTTTGGAAGCCCTGAAGCCAGAGCTTAAGGCCAGCATCGAGAAGGGGATCAATTTTGTTAACAAGCAAGCTGTGGCAGCGTAGAACTTCTACTGCACCTTCCGCATTTAGAAGAATAAATAAATCAATGATTTCAGTTTTGTAGGTTACTTGCCACAGAGGGCTTACTGCATCTTCACTTTGGTAATAGCCAAGTGAGTTTACCAAAACTTCTTCGTTTTCGATTTCTGTTACCATATGCGGCATTTTGTAGTGCACAAGGATATCATTGGAGCGCTAACTCCGTTAGATTTGCTCGGCTTTTGGATTAAGTGGTTTCTGTTCATCTCATATGTTCATGTGCTGAAATTTTGTCGATTAAAAAAAAAAAAAAAAAAAAAAAAA

14. phosphoenolpyruvate carboxykinase (ATP) (*PEPC*)

CAGAACGGCGGGTTCACGTTCGCGGCGAGTCCGACGATCGCGAGAGGTCGGAACGGACTTCCTAGGATCCACACGGAGAAGAAGGAGGAGGACGAGGTTTGCCACGATGACAGCGGGCCGGCGGTGAAGGTCCAGACGCTGGACGAGTTGCATTCCCTGCAGAGGAAGAAATCGGCTCCGACGACGCCGATCACCGGAACTCAGGGAGCCTTTGCCGCCGGCGTCGCCGACGATGAGCGCCAGAAGCAGCAGCTCCAGTCGATCAGTGCGTCGCTGGCGTCGCTGACTCGGGAAACGGGGCCGAAGGTGGTGCGGGGGGACCCGTCGAGGGCGGAGACGCCGCGGGTGTCGGCGGTGACGCACCACCATTACGAGCCGGCGTTCAGCATCAGTGACAGTGCGCTCAAGTTTACTCATATCCTTTACAATCTCTCACCGGCGGAGCTCTACGAGCAAGCAATCAAGTATGAGAAGGGATCCTTCATAACGGCGAGTGGCGCTTTGGCAACACTTTCTGGAGCAAAAACTGGTCGTTCTCCAAGGGATAAGCGCGTTGTAAGGGATGAGACCACTGAGGATGAGCTTTGGTGGGGAAAGGGTTCTCCCAACATTGAAATGGACGAACACACTTTCTTGGTTAACAGAGAGAGAGCCGTGGATTATCTGTGTTCCTTGGAGAAGGTTTATGTGAATGATCAGTTCCTTAACTGGGATCCTAACCACAGAATCAAAGTTAGGATTGTCTCTGCTAGAGCCTACCACTCATTGTTCATGCACAACATGTGTATCCGACCCACGCCCGAGGAGCTGGAGAACTTTGGTACTCCCGACTTCACGATATACAACGCCGGGCAGTTCCCATGCAACCGATACACGCACTATATGACTTCCTCCACTAGCATAGATGTGAATCTCGATAGGAAGGAAATGGTGATCCTTGGTACTCAGTATGCCGGGGAAATGAAGAAGGGTTTATTCAGCTTAATGCACTATCTCATGCCTAAGCGCGGGATCCTGTCCCTTCACTCGGGTTGCAATATGGGAAAAGGCGGAGATGTTGCCCTTTTCTTTGGACTATCAGGAACTGGAAAGACGACGCTTTCTACTGATCACAACAGATACTTAATTGGAGATGACGAGCATTGCTGGAGCGAGAATGGCGTGTCCAACATTGAGGGTGGTTGCTATGCTAAGTGCTTGATCTGGCAAGGGAAAAAGAGCCTGATATTTGGAATGCTATCAAGTTTGGCACTGTGCTGGAGAATGTTGTGTTTGATGAACACAGTCGAGAGGTGGACTACACCGACAAATCTGTAACGGAAAACACTCGAGCTGCATATCCGATCGAGTACATTCCCAATGCAAAAATACCTTGTGTTGGTCCTCACCCGAAGAACGTCATCCTCTTGTCTTGTGATGCATTTGGTGTCCTTCCCCCTGTCAGCAAACTGGACCTGGCTCAGACCATGTACCACTTCATCAGTGGCTACACAGCTGTGGTAGCAGGAACCGAGGATGGAATCAAGGAGCCTACTGCAACATTCTCAGCATGTTTCGGTGCAGCATTCATCATGCTTCACCCGACCAAGTATGCTGCAATGTTGGCCGAGAAGATGAAGAAACATGGAGCAACAGGATGGCTCGTGAACACCGGCTGGTCTGGTGGAAGCTATGGATCGGGAAGCCGTATCAAGCTGGCTTACACGAGGAAAATCATCGACGCGATTCACTCAGGGAGCCTTCTGGATGCGAAATACACTAAGACGGAAGTGTTTGGACTGGAGATCCCTACTGAGATTGAAGGAGTGCCTTCAGAGATTCTGGAACCTACCAACACTTGGTCCGATAAGAATGCATACAAGGAAACTATCCTGAAGCTGGGTGGTTTGTTCAAGAAGAATTTCGAGGTTTTTGCAAACTACAAGATAGGATCAGACAACAAGCTGATGGATGAGATTGTTGCTGCTGGTCCCAACTTC

15. F-box protein At1g67340 (*F-box*)

ATGAAAACAAGGGCCGGAATTTGCTATCCCAGAGTTTTCCGTGAGAAGATGTGTGCTGAGAAAGAGTACCTCGATGTATCGAACAAGAAGAGGCGGCGGGATGCGGCGGCGGATCCCGCCGGCGGTGGTTCACGGCGGAGCAAGCGGCGGCCGTTTGTTTCGCAAGAGCAGCCTGAGCTGTTTGAGGCTTTGCCGGATGATCTCGTCCTGTCTATTCTCTGCAAGCTCAGTTCCACCGCTACCTGTCCGGCTGATTTCGCTAACGTGTTACTGACGTGTAAGAGATTTAATGGATTAGGGCTGAATTCGATGGTGTTGGCTAAAGCGTCGCCGCAAATGTTGGCGTTGAAGGCGAATAAATGGTCGGAGAATGCTCACCGGTTTCTGAAGCAGTGTGCGGATTCCGGCAATGTTGAAGCTTGCTATACTCTCGGCATGGTGAGAAAATGATTATTTGTAATTAAAAATTATGTGATTATGGTTAAGGCAGTTGAATTTGAAGATTTTGTAGAGTTTGTAGTGTGAGAAACGGGTTATTAATGTGTGTGTGTAATGCAGATTCGGTTTTACTGTCTGCAAAACCGTGGAAGTGGAGCGTCTCTGATGGCGAAGGCGGCGATTAGCTCGCACGCGCCGGCGCTGTATTCACTCTCCGTCATCCAGTTCAACGGGAGCGGGGGGTCGAAGAATGATAAGGATCTACGCGCCGGTGTAGCTCTGTGCGCACGCGCCGCCTTCCTCGGCCACATCGACGCTCTCCGAGAGCTCGGCCATTGCCTGCAGGACGGTTACGGCGTGAAGCGGAACATTTCCGAAGGCCGGCGGTTTCTGGTCCAGGCGAACGCGCGTGAGCTGGCGGCGGTTCTGTCGGCGACGCCTTCCGCCTTACTCTCCGGGAACCTGCTGACGTGGAATGCACCGCCGGGCCACCAGCATGGCGGCGAGGCGGCGGCGCCCGGGTGTCCGCTGTTGAGTGATTTCGGGTGTAATGTGCCGGCGCCGGAAGCGCATCCGGCGAGCCGGTTTCTAACCGACTGGTATTCCGCCCGGGGCGGGAATCCGGGTCCGGGTCTGAGGCTGTGCTCTCATTCGGGTTGCGGCCGGCCGGAGACCCGGAAGCACGAGTTCCGGCGGTGCTCCGTCTGCGGCGCTGTGAATTACTGTTCACGCGCCTGCCAGGCGCTTGACTGGAAGATGCGGCATAAAGCTGAGTGCACTCCTGCTCAGCAA

16. o-succinylbenzoate-CoA ligase [*Rubia cordifolia*] (*OSBL*)

TRINITY_DN125216_c2_g1_i1

AGTGATACATACATGGAATGGGTCCTGGCAATCACGTGCGTGGGAGGAATAGCCGCCCCTTTCAACTACCGGTGGAGCGCCGAAGAGGCTCGGCAGGCCGCCAACGAGGTGAAGCCCGTCATGCTTGTCACCGACACCACCTGCGATCACTGGCACTCCACCCTGCAGCCCCACATCAGATGGCACGTCTCCATGAACCATGTCCGGCTCACCGACCCGACCTCCCTCAGTCCTCCCCGCCTTCTCTGGCCGCCCAACAAAGCTGCCCTCCTCTGCTTCACTTCAGGCACTTCCGGGAACCCCAAGGCTGCTGTTATAACCCATTCTGCTCTGGTCGTGCAGTCCCTTGCCAAGCTCGCGCTTGCTGGCTACACTGAGGATGATATTTACCTGCATACTGCTCCTTTGTGCCACATTGGGGGGCTATCATCAGCCCTGGCCAATTTGATGGCCGGCGCTCGCCATGTCATTCTACCCAAGTTTGAAGTTCAAGCAGCGGTCAAGGCCATGGAGGAGCACCATGTTACCTCTACTATCGTCGTGCCGGCCATGATGGCTGACCTCACCTCCACAACCAGGTCCATGAAACCTTCGAAGCAATTTCAGACGGTGCGGAAGATCCTAAATGGAGGCGGAGGCCTCTCAGCTGGCCTAATCCAGAGCGCTACCGAAACCTTCCCTAGGGCTAAACTAATGACTGCTTATGGCATGACAGAGGCATGCTCTTCTCTGACATTCATGACTCTGTACGATCCAACCTTCGGGAGCCACACTCAGCACCCTGAAATGATCAAGGGAAGAAGCTCGAGACTAGCCCAATCAGGCGGCGTGTGCGTAGGGAAGCCTGCACCCCATGTTGAAGTAAAAATAAGTCACGACGGTTCATCTGAAGGCGGCAAGATTCTGACCAGGGGACCTCACACAATGCTGGGATACTGGGGTCACACTCCAGCCAAGGCTTCGCGCCCCGAAGATGAGGGCTGGTTCGACACAGGTGATGTGGGAGCAATAGATGAGAATGGTAACCTTTGGCTCATGGGGCGCGACAGTGGTAGAATCAAGAGTGGAGGAGAAAATGTGTATCCTGAACAGGTAAAAATCCTCCTGTGAAGAAATGAAATCCTACATTTCTTTGTCTGATCCGTAACCTGTGTGAAAAAACAGGCAGAGGCCGTTTTATTGCAACATCCGGGAGTTGCTGCAGTGGTTGTCTTGGGGCTCCCGGACCCTCGCTTTACGGAGATGGTGGTTGCATGCATTCAACTGAAAGAAGACTGGAAGTGGGTTGACAGGGATGGCATGAATGACGGATCAGATAATTGCCTGTCCAAAGAAGTGCTTCAGCGCTTTTGTAAGCACAAGAACTTGACAGGGTAC

17. isochorismate synthase [*Rubia cordifolia*] (*ICS*)

TRINITY_DN125672_c2_g2_i4

GATGAGCTCGAAGGAAGCTCGAAGATGGCTGCAACAATCGCGTGGGACAGCGCGCTCTCTTGCTCGTATCAGAGCGCGATTGCTGAACTTAAATCAACAATGGCGAAGATTACATCTGCTCTTACAAGACAGCGAGTGGAGGCCTCTCATTCGGAGATAACAAGGAAGGCTCATGTTCCTAGCAGAACAGATTGGGATGTCACAGTTAATAAAGCTTTGGACAGTATCAAATCAGCTGATTCGCCATTGACAAAGGTTGTTCTTGCGCGAAGCAGCCAGGTTTTGACCTCGGATGAAATCGATCCCTTAACATGGCTGGATACATTGAAGGGCGAAGGAAACGAGTCTTATCAGTTCTGCCTTCAGCCTCCGGATTCAGCAGCTTTCATTGGAAACACACCTGAACAGCTCTTTCGCCGCGATCAGTTGACTGTTTTCAGTGAAGCATTGGCTGCGACCCGAGCTAGGGGTGCAACGCCGTCCACGGATCTTCAGATTGCTCAAGACTTATTTTCCAGCACTAAGGATCACCATGAATTCGCTATCGTGCGAGAGAACATCAGGGGAAAGCTGAACGCTGCGTGCACGAACGTGCAAGCTAATCCGGAGAAGGTTGTTCGCAAACTGGCCAGAATTCAGCATCTTTATGGCCGATTTTCGGGTACATTGAACTCTGCAGATGATGAGTTCACGATCTTATCATCCCTTCATCCAACTCCAGCAGTTTGCGGTTTTCCTGCTGAAGATGCGAGAAAATTCATCACAGATAACGAAATGTTTGATCGGGGGATGTACGCCGGCCCTGTTGGTTTCTTTGGCGGCGCTCAGAGTGAGTTCTCTGTCGGAATAAGATCTGCCTTAATTGGAAAGGATATTGGAGCCCTAATCTATGCTGGACTTGGCGTGGTAGAAGGAAGCGTTCCTTCGCTAGAATGGGAAGAGCTCGAGCTGAAAGCATCGCAGTTCATGAAGCTGATGAAGTTAGAGGCGCCGGCATTGGCAGCAATAGCATAGAGAAAATAATGCCCTAGTTAGAGATACTTTGAAGAATAAAAGCAATATTACCACTTAAAATGCCATTATTCAGCCTAAGAAGACTGTGAAATTGAAGGCAAAGATGGTGATATTTACCGGAATGTAGACATATCTTTTTTCTTTCATTATTTTAGTGTTCGGGGTAATGTTTGTATAGAGAAAATCACAGCCTCCATAGTATCATTCTTGGCTTTCTTTCTGTTCAAATTTGTTCATCATTTTGAGGCTTAGATTTGGGTTTGAAGTTGTAAGCTCTCAAACTCTTGTAAAAAAAATCCGTACAT

18. isopentenyl-diphosphate delta-isomerase [*Rubia cordifolia*](*IPPI*)

TRINITY_DN131965_c2_g1_i8

CAGAGGCGCCTCATGTTCGAGGATGAATGTATTCTGGTAGACTCATATGATCATGTTGTT

GGCCATGACAGCAAATACAATTGTCACTTGATGGAAAAGATTGAATCTGAAAATTTGCTGCATAGAGCTTTCAGTGTGTTCTTATTCAACTCAAAGTTTGAGTTGCTGCTTCAGCAACGTTCTTCAACAAAGGTAACCTTTCCGTTGGTCTGGACAAATACCTGTTGCAGCCATCCTCTTTTCCGAGAATCTGAACTTATTGAAGAGAATAGTCTTGGAGTGAGAAATGCTGCTCAGAGGAAACTTTTTGATGAACTGGGTATTCCAGCTGAAGATGTTCCAGTTGATCAGTTCACCCCCTTGGGCCGTATTCTCTACAAGGCCCCATCCGATGGAAAGTGGGGAGAGCATGAACTTGACTACCTACTCTTCATTACCCGGGATGTCAAGGTGAATCCCAATCCTGATGAAGTTGCTGAGATCAAGTATGTCAACCGAGACGAGTTGAGGGAGCTTTTAAGAAAGGCAGATGCCGGTGAGGGTGGCCTGAAGCTGTCTCCATGGTTCAGACTAGTTGTTGACAACTTTTTGTTCAAGTGGTGGGATCATGTTGAGAAGGGAACCCTGACTGATGCTATTGACATGAAAACCATTCACAAG

19. 3-hydroxy-3-methylglutaryl-coenzyme A reductase[*Coffea canephora*](*HMGR*)

TRINITY_DN129754_c4_g1_i1

GATGCGCTGCCGCTCCCTCTCTACCTCACCAACACCGTCTTCTTCACCCTCTTCTTCTCT

GTCGCCTACTTCCTCCTCCACCGCTGGCGCGACAAGATCCGCACCAACACCCCGCTCCACGTCCTCACTCTTTCCGAGCTCGCCGCCATTCTCTCCCTCGTCGCCTCCTTCATCTACCTCCTCGGATTCTTCGGCATTGAATTCGTCCAGTCCTCCGCCCCCTCCCGCCCTTCCTCCGGCGACCCCTGGGACGACGTCGTTCCCATCGACCACCCCCGGCCTGGCTTTCCCCCCGTTCCAATGGAGCGACAGCATGTGGATATGGACCTCGACGATGAGAGCGAGGAGCTCGTAAACGCCGTCGTTTCGGGCCGTGCGCCGTCGTACTCGCTCGAATCCACCACCGGCGATTGCTTCAAGGCGGCGTCGATTCGGCGGGAGGCCGTGCAGAGGATCACCGGGAGATCTCTCGCCGGGATGCCTCTCGAGGGGTTCGACTACGACTCCATCTTAGGGCAGTGCTGCGAGATGCCCGTCGGCTACGTCCAGATTCCGGTGGGAGTCGCGGGGCCGCTGCTGATGAACGGTTTCGAATACTCCGTCCCCATGGCCACCACGGAGGGGTGCTTGGTAGCCAGTACGAACAGGGGATGCAAGGCCATTTTCGCCTCCGGCGGGGCCACCAGCATTCTCCTCAGAGACGGGATGACAAGAGCTCCGGTGGTCAGGTTCCCCACCGCCAAAAGGGCCACTCTTTTGAAGTTTTTCTTGGAGGATCCTCTCAATTTCGAAACCATCTCCCTCATGTTCAACAAGTCTAGCAGATTTGCTAAGCTTCAGGGCATTCAGTGCTCCATTGCTGGCAAGAATCTGTACATCAGGTTTAACTGCAGCACTGGAGATGCCATGGGGATGAACATGGTTTCCAAAGGTGTTCAGAACGTCCTTGACTTCCTACTTACTGAATTCCCAGACATGGATGTCATTGGCATTTCTGGGAATTTCTGTTCTGACAAAAAACCTGCTGCAGTGAATTGGATAGAAGGGCGTGGGAAATCTGTTGTGTGTGAGGCGATTATCAGCGAAGAGGTGGTGAGGAGTGTTTTGAAAACCACGGTGCCAGCTCTGGTAGAGCTCAACATGCTCAAGAATCTCACTGGTTCTGCTATGGCTGGAGCTCTGGGTGGTTTTAATGCGCACGCCGCGAATATCGTGTCTGCCATTTTCATTGCAACCGGACAGGATCCAGCGCAGAACATAGAAAGCTCCCACTGTATCACTATGATGGAGGCTGTGAACAATGGCAAGGATCTCCACGTTTCCGTCACTATGCCTTCCATTGAGGTCGGGACAGTAGGTGGTGGAACTCAACTAGCATCTCAGTCGGCTTGCCTGAACTTGCTGGGAGTGAAAGGGGCGAATGCAGAATCGCCGGGATCAAATGCTCGGCTGCTGGCCACCATAGTGGCCGGAGCAGTACTGGCAGGGGAGGTGTCTCTGATGTCGGCCATAGCGGCAGGGCAGCTGGTTAAGAGCCACATGAAATACAACAGATCATCCAGGGACGTCACTAAGATT

20. 1-deoxy-D-xylulose 5-phosphate synthase [*Gardenia jasminoides*](*DXS*)

TRINITY_DN124410_c3_g1_i1

ATTGCCGTAAACCAGTCGACATCTCCCTACGCCGCCGCCCCACGCCTCTGTTCCGCCGCAAGAAAACAGTTTTGCCTGAGGGCTTCCGCAAGTAGTAATAACAGCCCCGATGGCGGTGATGAGGGGAAGATGGTGATAAAGAAGGAGAAAGACGGGTGGAAGGTTGATTTCTCCGGGGGAAAGCCTCCGACTCCACTGCTGGACACCATCAACTACCCTATTCACATGAAAAACCTCACCGTACAGGAGCTTGAGAGATTGGCGGCGGAACTGAGAGCGGAGATCGTGTACGGCGTTGCGAAGACGGGTGGGCATCTGAGCTCGAGCCTGGGAGTAGTGGAGTTGACTATTGCCCTGCACTACGTTTTCAACACTCCAGATGACAGGATCATTTGGGATGTCGGCCATCAGGCGTACACGCATAAGATTCTCACCGGAAGAAGGGCTAAAATGCAATCCATCAGGCAGACCTATGGGATCGCGGGCTTTCCCAAGAGGGACGAAAGCGTCTACGATGCTTTCGGCGCCGGCCACAGCTCCACCAGCATTTCTGCCGGACTCGGTATGGCGGTGGGAAGAGATCTTCTTGGCAAGGACAACAACGTTATCTCTGTCATTGGAGATGGCGCCATGACGGCCGGACAAGCTTACGAAGCCATGAACAACGCGGGATTCCTTGATTCCAACCTTATTGTCATACTGAACGATAACAAGCAGGTTTCTCTGCCTACGGCTACTCTTGATGGGCCTGCGACCCCGGTTGGAGCGCTCAGCAGTGCTTTGACCAAGCTCCAAGCTAGTCCAAAGTTCAGGCAACTTCGCGAGGCAGCGAAAAGTTTGACCAAGCAAATTGGACCACAAGCACATGAAGTTGCTGCCAAATTCGATGAATATGCTAGGGGAATGATAAGTGCTTCTGGATCTACTTTCTTTGAGGAGTTGGGATTGTACTATATTGGACCAGTGGATGGGCACAATCTTGAAGATCTGGTCACCATTTTCGAGAAGGTTAAATCTATGCCAGCTCCAGGGCCGGTACTAATTCATCTTGTGACGGAAAAGGGAAAAGGCTATCCGCCCGCCGAGGCTGCAGCTGATAAGATGCATGGAGTTGTCAAGTTTGATGTGAAAACAGGGAAGCAACTCAAGAAAGCAGCTCCTACGCTTTCATACACGCAGTACTTTGCTGAAGCCCTGATCAAAGAAGCTGAAATTGATAACAAAATTGTGGGCATCCATGCTGCCATGGGAGGTGGAACTGGTCTCAACTATTTCCAGAAGCAGTTTCCAGAACGGTGCTTCGATGTGGGGATCGCTGAGCAGCACGCTGTTACATTTGCTGCGGGTTTGGCCACAGAAGGCCTGAAGCCATTCTGTGCAATCTACTCCTCATTCCTTCAAAGAGGATATGATCAGGTGGTGCATGATGTAGATCTTCAGAAATTGCCAGTGAGATTTGCCATGGACAGAGCAGGTCTAGTGGGTGCAGATGGACCAACTCACTGTGGTGCATTTGATGTTGCATATATGGCTTGCTTGCCAAACATGATAGTCATGGCGCCAGCCGACGAAGCAGAACTCATGCACATGGTAGCAACAGCAGCAACAATCGACGATAGACCATCATGCTTCAGGTTCCCGAGGGGCAATGGCATTGGTGTCCCTCTTCCTCCCGGCAACAAAGGAGTCCCTATCGAGATTGGAAAAGGAAGAATCTTGCAAGAAGGCAGCAGAATCGCAATCATTGGATATGGTTCTATAGTGCAAGAATGTGTTGGTGCTGCGGAAATGCTGAAATCCCAGAACATCACACCCACTATTGCCGATGCAAGATTCTGCAAACCGTTGGACGGTGATCTTATCAAAAGACTGGCTAAGGAGCATGAGATCTTAATCACAGTTGAGGAAGGTTCCATTGGAGGCTTTGGATCCCACGTAGCTCATTTCCTGAGCTTAAATGGCATTCTGGATGGACCCCTTAAGTTGAGATCAATGGTACTCCCAGATCGATACATCGACCACGGAGCACCAGCGGATCAG

ATTGAACAAGCGGGTCTATCTTCAAAGCACATTTCTGCAACCGTTCTTTCATTGCTCGGAAAACCCCAGGAAGCTCTTCAATTCATATAAACTTTTGCATTCAAGTTACAACGGTTCCACAACACGTTATCTATGAATTGCTTCAATATAGAGCCCCTTCTATAGGGTAGGAAGAAAGGACACGTACTCCAATCAAAGAATCCGAAATTTCTGATGAGAAGGTAGGGATTCTGGATAGGCATTAATGCAGCTTTTGGAACTCATGTGTCAAAAGGAATGTTCATCTCCTAGGCATAATATGTACTGTTATGTAACTGTATCCTTCAGATTTTGTGAATAAGTGTAACAATAAGATGGTTGTTTGATTGTTTTGATTTCAATTGTTTGTTCTTATTGTCCTGATGTGATGAATTGACAGGGGTAAAGAAACTGGTAATGATGTTAGA

21. acetyl-CoA acetyltransferase[*Coffea canephora*](*AACT*)

TRINITY_DN130086_c0_g2_i13

ATGGCTCCCACTGCAGCATCAGAGAACTCAATAAAACCAACAGATGTTTGCATTGTTGGTGTTTCCCGTACCCCAATGGGTGGTTTCCTGGGCACACTTTCATCTTTACCAGCAACAAAGCTTGGATCTATAGCCATCGAGGGTGCACTCAAGAGGGCAAAAGTTGATCCATCACTCGTGCAAGAAGTTTTCTTCGGGAATGTTCTCAGCGCAAATCTGGGGCAGGCTCCAGCTAGGCAGGCAGCAATTGGTGCTGGGATCTCAAATTCAGTTGTTTGTACTACCATTAATAAAGTTTGTGCCTCTGGGATGAAAGCAACCATGCTTGCAGCACAGAGTATCCAGTTGGGCATTAACGATATTGTTGTAGCTGGTGGCATGGAAAGCATGTCAAATGTTCCCAAATACATAGCAGAAGCAAGGAAGGGATCTCGACTTGGACATGACACACTTGTGGATGGAATGTTAAAAGATGGACTATGGGATGTTTATAATGATCAAGGAATGGGAGTTTGTGCTGAATTGTGTGCTGAAAATCACAGCATAACTAGAGATCAACAGGATGATTATGCAATTCAGAGCTTTGAGCGTGGTATTGCTGCTCAAACTGCCAGTGCCTTCTCATGGGAGATTGTGCCTGTTGAAGTGTCTGGTGGAAGAGGCAAACCCTCTACTACAGTTGATAAAGATGAAGGCCTTGGAAAGTTTGATGCTGCAAAGTTAAGGAAGCTTCGTCCTAGCTTCAAGGAAACTGGAGGCAGTGTTACTGCAGGAAATGCTTCCAGCATTAGTGATGGTGCTGCCGCTCTAGTCTTGGTTAGCGGAAAGAAGGTTGTTGAGCTTGGGCTAGACGTGATTGCTAAGATAACAGGATATGCTGATGCTGCTCAGGCACCAGAGTTATTCACAACCGCGCCTGCTCTAGCAATTCCAAAAGCTATTTCAAATGCTGGCCTTGATGCATCAAAGATTGATTTCTATGAAATCAACGAAGCATTTGCGGTTGTGGCTCTTGCAAACCAGAAGCTTCTCGGGCTTAATCCCGAAAAGGTCAATGTTCATGGAGGGGCTGTATCACTGGGGCATCCTCTTGGGTGCAGCGGCGCCCGTATCTTAGTTACACTTTTGGGGGTATGTTACCATTTTCATTGTATTACTTGTAATGTATTAAGTGCACGGATCATGATTTCTCGAACCTTGAGAAACTAGAAAAGTGAGTGGTGGGAATGCTGTTTTCGGGTACTCATTCGGGTTTCCTACCCATTTCTTGCAGGTTCTGAGGCAGAAGGGCGGCAAAGTAGGCGTTGGAGGTGTTTGCAACGGAGGGGGAGGCGCTTCTGCTCTAGTCCTAGAACTCGTGTAATTCCATTTTTTTTGTGATTGTGTTAGAATGGAGCAACATTAGTGTAACTGGTGAGATGTTGAGTTTGCAGAAAATATGTCGTAATGAGGGAGAGAAAGATGACTTTGTTTAAGGCCTAGGAGATGCTACAAGGGGCTTTGTGTGTGTGATGTCTTTTGTTTCACTTGTACTTTACCATCAATAATGCAATTAATTAATTTTTTTTTTTTTTTTTTTTTTTTTTTTTTTTTTTTTTT
